# Comparisons of acupuncture therapies combining conventional treatment in the management of vascular cognitive impairment: a systematic review and network meta-analysis

**DOI:** 10.3389/fnagi.2025.1559388

**Published:** 2025-06-16

**Authors:** Yuan-Ling Liao, Pei-Shan Hsu, Chang-Ti Lee, Li-Jen Su, Yi-Ying Shen, Adam Tsou, Chou-Chin Lan, I-Shiang Tzeng, Guan-Ting Liu, Po-Chun Hsieh

**Affiliations:** ^1^Department of Chinese Medicine, Taipei Tzu Chi Hospital, Buddhist Tzu Chi Medical Foundation, New Taipei City, Taiwan; ^2^Department of Biomedical Sciences and Engineering, National Central University, Jhongli, Taiwan; ^3^Institute of Systems Biology and Bioinformatics, National Central University, Jhongli, Taiwan; ^4^Stroke Center and Department of Neurology, Taipei Tzu Chi Hospital, Buddhist Tzu Chi Medical Foundation, New Taipei City, Taiwan; ^5^Division of Pulmonary Medicine, Taipei Tzu Chi Hospital, Buddhist Tzu Chi Medical Foundation, New Taipei City, Taiwan; ^6^School of Medicine, Tzu-Chi University, Hualien, Taiwan; ^7^Department of Research, Taipei Tzu Chi Hospital, Buddhist Tzu Chi Medical Foundation, New Taipei City, Taiwan; ^8^School of Chinese Medicine, National Yang Ming Chiao Tung University, Taipei, Taiwan

**Keywords:** vascular dementia, vascular cognitive impairment, acupuncture therapy, systematic review, network meta-analysis

## Abstract

**Background:**

Vascular cognitive impairment (VCI) is the second most frequent form of cognitive disorder. It is mainly caused by a diseased cerebral vasculature and affects patients’ cognition and activities of daily living (ADL). Previous studies have demonstrated that acupuncture therapy is a promising complementary treatment that significantly improves cognitive status and ADL in VCI patients. This study aimed to investigate the effects of different types of acupuncture therapies and conventional treatments on cognitive status and ADL in VCI patients to provide evidence-based clinical recommendations.

**Methods:**

We searched seven electronic databases for randomized controlled trials comparing acupuncture therapies [including manual acupuncture (MA), scalp acupuncture (SA), electroacupuncture (EA), and auricular acupuncture (AA)] with conventional treatment [pharmacotherapy (P), cognitive rehabilitation (CR)] or standard care (SC) in patients with VCI. The primary outcome was cognitive improvement, while secondary outcomes included improvement in ADL and the risk of severe adverse effects. A frequentist random-effects network meta-analysis was performed under a consistency model. Study quality was assessed using the RoB 2.0 tool. Inconsistency was examined via node-splitting. Subgroup analysis, meta-regression, and sensitivity analysis were conducted to explore heterogeneity and assess robustness. Publication bias was evaluated using funnel plots and Egger’s test.

**Results:**

Through stepwise exclusion of studies contributing to publication bias and inconsistency, a robust bias-adjusted network meta-analysis dataset was established. The results showed that among all interventions, SA+P+SC demonstrated the greatest efficacy in improving cognitive status compared to SC (SMD: 2.04; 95% CI: 1.21–2.86) with substantial heterogeneity (I^2^ = 71.0%), no significant inconsistency, and relative low publication bias (*p* = 0.7020).

**Conclusion:**

Acupuncture, particularly SA combined with P and SC, appears to be a safe and effective adjunctive treatment for patients with VCI. Future studies are warranted to establish VCI-specific MCID thresholds and to validate these findings through large-scale, high-quality RCTs.

**Systematic review registration:**

https://inplasy.com/inplasy-2023-5-0114/, identifier INPLASY202350114.

## Background

Vascular cognitive impairment (VCI) refers to a spectrum of cognitive disorders primarily caused by cerebrovascular disease or impaired cerebral blood flow. It encompasses a range of symptoms, from subjective cognitive complaints to various stages of dementia, including vascular dementia (VD), multi-infarct dementia (MID), post-stroke cognitive impairment (PSCI), and vascular cognitive impairment no dementia (VCIND) (van der Flier et al., 2018; Graff-Radford, 2019). VCI is a progressive condition characterized by declines in memory, cognition, language, behavior, and executive function (Zhao et al., 2009). Its prevalence increases with age, affecting approximately 1.6% of individuals over 65 years old (Sarfo et al., 2017), and significantly reduces patients’ quality of life and increases caregiver burden (Lobo et al., 2000). According to the Global Burden of Disease (GBD) 2021 study, dementia accounted for one of the highest disability-adjusted life year (DALY) burdens among neurological disorders, with DALYs in individuals under 65 increasing by nearly 120% since 1990, underscoring its growing impact on global health (Li et al., 2024).

As for the vascular etiology of VCI, prevention strategies focus on managing modifiable risk factors such as hypertension, diabetes, hyperlipidemia, and smoking (Rundek et al., 2022). Current treatments include pharmacological agents (e.g., donepezil, galantamine, rivastigmine, and memantine) and non-pharmacological approaches such as cognitive rehabilitation (CR), behavioral therapy, psychological support, and caregiver education (Rundek et al., 2022). However, the efficacy of pharmacotherapy in VCI remains modest, with limited impact on global cognition or daily function (Farooq et al., 2017). CR is commonly used to manage VCI, but its effectiveness remains limited. A Cochrane review found no strong evidence supporting its effectiveness in improving cognition, mood, or daily function in patients with Alzheimer’s disease (AD) or VD (Bahar-Fuchs et al., 2013).

With these limitations, there is a growing interest in exploring complementary therapies, such as acupuncture, which may offer additional benefits in managing cognitive symptoms associated with VCI. In Traditional Chinese Medicine (TCM), acupuncture remains a widely used therapeutic approach for both prevention and treatment of various physical and neurological disorders. Recent reviews have highlighted its applications across stroke rehabilitation (Yang A. et al., 2016; Huang et al., 2022), neurodegenerative diseases (Tan et al., 2024), and cognitive disorders secondary to cerebrovascular disease. Han et al. (2021) reported that early acupuncture improved cognitive function and activities of daily living (ADL) in VD patients after cerebral infarction by enhancing cerebral blood flow and perfusion. Zhang S. Q. et al. (2024) showed that combining scalp-abdominal acupuncture with donepezil produced greater cognitive and functional gains than donepezil alone in patients with AD. Similarly, Zhan et al. (2016) demonstrated that electroacupuncture at Baihui (GV20) and Shenting (GV24) improved quality of life in VCIND patients.

Previous pairwise meta-analyses have suggested that acupuncture may be a promising intervention for patients with VCI. Su et al. (2021) demonstrated that acupuncture is beneficial for improving both cognitive function and activities of daily living in VCI patients, with minimal adverse effects. Similarly, Cao et al. (2013) reported that acupuncture has a positive effect on cognitive and memory functions in individuals with mild VCI. These findings highlight the potential role of acupuncture in the clinical management of VCI. Building on this foundation, several recent network meta-analyses have further explored the comparative efficacy of acupuncture and other non-pharmacological interventions. Wen et al. (2022) focused on different acupuncture modalities in VD and found that combined treatments such as moxibustion with body acupuncture (MB+BA) and electroacupuncture with scalp and body acupuncture (EA+SA+BA) were more effective than single-technique approaches. Li et al. (2023) investigated patients with vascular cognitive impairment with no dementia (VCIND) and identified manual acupuncture combined with Chinese herbal decoction as the most effective for improving cognitive function. Yi et al. (2024) evaluated a wide range of non-pharmacological therapies for VD, concluding that the combination of acupuncture, moxibustion, and conventional treatment was among the most efficacious interventions for enhancing both cognition and daily function.

However, most existing studies either focused exclusively on different acupuncture modalities or lacked direct comparison with conventional treatments widely adopted in clinical practice, including P, CR, SC. As these standard interventions remain the foundation of VCI management, it is crucial to assess how various acupuncture techniques may function as adjunctive therapies. Such comparisons are essential for developing integrated, evidence-based treatment strategies.

To address these gaps, the present study aimed to conduct a network meta-analysis to compare the efficacy of four acupuncture modalities, manual acupuncture (MA), electroacupuncture (EA), auricular acupuncture (AA), and scalp acupuncture (SA), when used in combination with P, CR, or SC, in improving cognitive function among patients with VCI.

## Methods

### Study design

This study was conducted in accordance with the Preferred Reporting Items for Systematic Reviews and Meta-Analyses (PRISMA) extension guidelines for network meta-analysis (Hutton et al., 2015) and the Cochrane Handbook for Systematic Reviews of Interventions (Higgins et al., 2024). A study protocol was created and registered in the International Platform of Registered Systematic Review and Meta-analysis Protocols (INPLASY; registration number: INPLASY202350114). Two reviewers (YLL and PSH) independently conducted database search, study selection and data extraction followed the determined protocol, and discrepancies were resolved by a third reviewer (PCH).

### Search strategy

We searched seven electronic databases: PubMed, Embase, Cochrane Central Register of Controlled Trials (CENTRAL), China National Knowledge Infrastructure (CNKI), Airiti Library, WanFang, and VIP. Relevant articles were searched without language restrictions from inception until 31 March 2025.

The following keywords were used in search of English databases: [“Dementia, Vascular” (MeSH) OR “vascular cognitive impairment”] AND (“acupuncture” OR “manual acupuncture” OR “scalp acupuncture” OR “electroacupuncture” OR “auricular acupuncture” OR “fire needling” OR “warm needling”). We also used keywords in Chinese synonyms to search Chinese databases. Detailed definitions of the PICOS are listed in Supplementary Table 1. Full details of the search strategies and results are presented in Supplementary Table 2.

### Study selection criteria

The analysis included only RCTs. The population comprised patients diagnosed with VCI [including multi-infarct dementia (MID), post-stroke cognitive impairment (PSCI), VCI with non-dementia (VCIND), and vascular dementia (VD)] based on established and validated diagnostic definitions (e.g., DSM-IV). All retrieved studies had at least two comparative treatment arms, one arm with a type of acupuncture intervention such as MA, EA, SA, AA, fire needling, or warm needling, while the other included conventional treatment, including pharmacotherapy (P, such as donepezil, galantamine, rivastigmine or memantine), cognitive rehabilitation (CR), or standard care (SC, defined as the management of vascular risk factors such as hypertension, hyperlipidemia, diabetes, and smoking cessation) (Table 1).

**TABLE 1:** The definitions and abbreviations of the treatments.

Treatment	Abbreviations
Auricular acupuncture	AA
Electroacupuncture	EA
Manual acupuncture	MA
Scalp acupuncture	SA
Cognitive rehabilitation	CR
Pharmacotherapy	P
Standard care	SC

Studies were excluded if they met any of the following criteria: (a) included patients with Alzheimer’s disease or cognitive impairment not meeting VCI criteria; (b) included patients diagnosed with depression, other psychiatric disorders, or severe neurological impairments that could interfere with neuropsychological assessments; (c) duplicate publications or studies with overlapping data; (d) non-RCTs, including meta-analyses, reviews, theoretical discussions, clinical observations, and animal studies; (e) studies for which the full text was unavailable, or the means and standard deviations (SD) could not be extracted, obtained or request from the authors; (f) studies that did not report at least one primary or secondary outcome relevant to the analysis; (g) studies used herbal medicines or moxibustion, or did not specify acupuncture points.

### Data extraction

We censored all the retrieved articles and extracted data using a predetermined form. The following information was recorded: author, year, diagnostic criteria, patient age, sex, sample size, intervention arms, outcome measurements, acupuncture point formula, and acupuncture-related treatment variables: needle retention time (in minutes), weekly treatment frequency (sessions per week), and treatment course (in weeks).

The outcome measurements with continuous variables were extracted as mean ± SD. For studies in which the data were expressed as 95% confidence intervals (CIs) or interquartile ranges (IQRs), we performed mathematical transformations according to the recommendations of the Cochrane Handbook (Higgins et al., 2024). When a 95% CI was reported, the SD was calculated using the formula: SD = $\sqrt{N}$ (upper limit–lower limit)/3.92. The width of the IQR was assumed to be approximately 1.35 times the SD. In the present study, all data extracted from the included studies were ultimately presented as mean ± SD.

### Outcome measurements

The primary outcome was the cognitive status, measured by the Mini-Mental Status Examination (MMSE), Hasegama’s Dementia Scale (HDS), Montreal Cognitive Assessment (MoCA), or Alzheimer’s Disease Assessment Scale-cognitive subscale (ADAS-cog). The outcome values before and after the intervention and the mean differences were recorded if available. The mean differences represent cognitive and ADL improvements after the intervention. The secondary outcome included: (1) the activities of daily living (ADL), measured by the Activities of Daily Living Scale (ADLS), Barthel Index (BI), or Functional Activities Questionnaire (FAQ); (2) the risk ratio (RR) of the presence for severe adverse effects. The severe adverse effects of acupuncture therapy include organ injury such as pneumothorax, cardiac tamponade, central nervous system damage, or abdominal organ perforation; infections including bacterial or viral transmission; peripheral nerve injury; broken needles requiring surgical removal; severe bleeding especially in anticoagulated patients; and systemic reactions such as syncope or shock (Xu et al., 2023).

### Network meta-analysis

To compare the relative effectiveness of all interventions, a frequentist random-effects network meta-analysis was conducted under the assumption of consistency across the treatment network. This approach allows for the simultaneous comparison of multiple treatments by integrating both direct (head-to-head) and indirect evidence from all included studies. The random-effects model was chosen to account for potential heterogeneity across studies, acknowledging that the true treatment effects may vary due to differences in populations, interventions, and study designs. The statistical analyses were implemented using the package netmeta (ver. 3.2-0) in RStudios (ver. 2024.12.1+563) (R Core Team, 2020), which estimates summary effect sizes, 95% confidence intervals, and generates network plots and ranking probabilities. We used P-score for treatment ranking (Rücker and Schwarzer, 2015). Graph generation were performed using the packages netmeta (ver. 3.2-0), ggplot2 (ver. 3.5.2), and reshape2 (ver. 1.4.4) in RStudios (ver. 2024.12.1+563) (R Core Team, 2020).

### Inconsistency assessment

To evaluate the consistency assumption underlying the network meta-analysis, we employed the node-splitting model. This approach compares direct and indirect estimates for each treatment comparison separately, allowing for the identification of discrepancies between the two sources of evidence. A significant difference (*p* < 0.05) between direct and indirect estimates was interpreted as evidence of inconsistency. The node-splitting analysis was performed using the netsplit() function from the netmeta package in RStudio, which provides a formal statistical test for inconsistency at each comparison node within the treatment network.

### Publication bias

To evaluate the potential presence of publication bias, a comparison-adjusted funnel plot was constructed based on the standardized mean differences (SMDs) derived from the network meta-analysis. Visual inspection was complemented by Egger’s regression test to detect asymmetry, with a significance threshold of *p* < 0.05. Studies that appeared in the lower right quadrant of the funnel plot (indicating disproportionately large effect sizes and high standard errors) were considered at high risk of contributing to publication bias.

### Sensitivity analysis

To assess the robustness of the findings, a sensitivity analysis was performed by excluding studies identified as high risk for publication bias. The network meta-analysis was repeated using the remaining studies to determine whether the overall treatment effect estimates, and relative treatment rankings were substantially affected. This approach aimed to confirm the stability and reliability of the primary results under different assumptions regarding data quality.

### Subgroup analysis

With the heterogeneity among patients with VCI, a subgroup analysis was conducted based on specific diagnostic subtypes reported in the included studies. These subtypes included vascular dementia (VD), multi-infarct dementia (MID), vascular cognitive impairment no dementia (VCIND), and post-stroke cognitive impairment (PSCI). The purpose of this analysis was to explore whether treatment effects varied across different VCI subtypes and to assess the robustness of the main findings within more homogeneous clinical populations. Subgroup analyses were predefined and conducted using the same network meta-analysis model, with treatment effects estimated separately within each diagnostic category.

### Meta-regression analysis

To investigate the influence of study-level covariates on treatment outcomes, meta-regression analyses were performed. Prespecified covariates included participants’ mean age and key acupuncture-related treatment parameters: needle retention time (minutes), weekly treatment frequency (sessions per week), treatment course (weeks), total number of treatment sessions, and cumulative treatment duration (total minutes across all sessions).

Effect sizes were calculated as SMD in cognitive score improvement, with variances derived from reported standard deviations and sample sizes. Weighted least squares (WLS) meta-regression was performed using inverse-variance weighting to account for study precision. Regression coefficients (β), 95% confidence intervals, R^2^ values, and *p*-values were reported. Analyses were conducted in Python (v3.11.6) using statsmodels, pandas, numpy, and matplotlib for data processing and visualization.

### Risk of bias assessment

Risk of bias was assessed using the Cochrane Risk of Bias 2.0 (RoB 2.0) tool (Sterne et al., 2019) across five standard domains and an overall judgment. The evaluation was conducted independently by two reviewers (YLL and PSH), with discrepancies resolved through consultation with a third reviewer (PCH). To visually summarize the domain-level assessments for each included study, a traffic light plot and a stacked bar chart of domain-wise risk distributions were generated. The traffic light plot displays individual study judgments across all domains using color-coded markers (green = low risk, yellow = some concerns, red = high risk), while the horizontal stacked bar chart summarizes the proportion of studies falling into each risk category by domain, including the overall risk of bias. Visualizations were created using Python (ver. 3.11.6), using matplotlib, seaborn, pandas, and numpy.

### CINeMA assessment

The certainty of evidence was assessed using the CINeMA (Confidence in Network Meta-Analysis) web application^1^ (Nikolakopoulou et al., 2020), which operationalizes the GRADE framework for network meta-analysis (Puhan et al., 2014). We evaluated six domains: within-study bias, reporting bias, indirectness, imprecision, heterogeneity, and incoherence. Risk of bias was assessed using the Cochrane Risk of Bias 2.0 tool for each included study. Each domain was rated as having “no concerns,” “some concerns,” or “major concerns.” An overall confidence rating was then assigned based on the level and number of concerns, categorized as high, moderate, low, or very low. As for the imprecision domain, due to the absence of an established minimal clinically important difference (MCID) or minimal clinically important difference (MID), we applied a surrogate threshold of ± 0.5 SMD to define the zone of no important effect, as recommended by the CCCG Supplementary author advice of Cochrane Handbook (Ryan and Hill, 2016).

## Results

### Study identification

**FIGURE 1:**
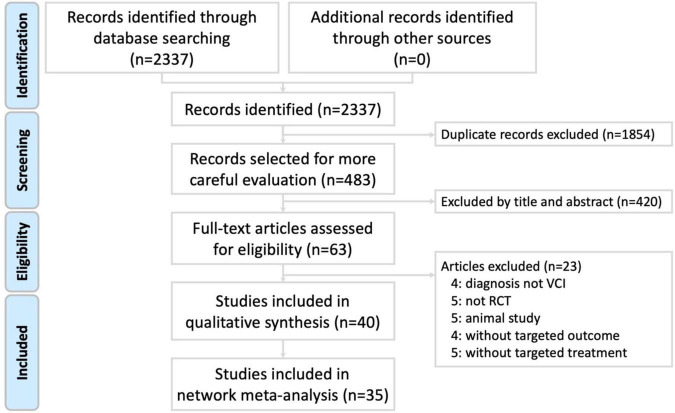
Preferred reporting items for systematic reviews and meta-analysis (PRISMA) study flow diagram.

The review process was presented as a PRISMA study flow diagram (Figure 1). We selected 2,337 studies using search terms in seven databases: 176 in PubMed, 305 in Embase, 73 in Cochrane Central, 646 in the China National Knowledge Infrastructure (CNKI), two in the Airiti Library, 496 in the WangFang and 639 in the VIP database. After eliminating 1,854 duplicate studies and 420 studies by titles and abstracts, 63 studies were retrieved for full-text evaluation of eligibility. Subsequently, 23 articles were excluded (four in which VCI was not diagnosed, five that were not RCTs, five animal studies, four without targeted outcomes, and five without targeted treatments; Supplementary Table 3). Finally, 40 RCTs were included in the risk of bias assessment and network meta-analysis (Mo et al., 2000a,b; Niu, 2007; Chen et al., 2009; Meng et al., 2009; Zhao et al., 2009; Yin et al., 2011; Li P. et al., 2012; Li S. et al., 2012; Li W. et al., 2012; Lin et al., 2012; Teng and Lai, 2012; Zhao, 2013; Cao et al., 2014; Li and Jiao, 2014; Li et al., 2014; Cui et al., 2015; Luo et al., 2015; Zhang et al., 2015; Yang W. H. et al., 2016; Tan et al., 2017; Wang, 2017a,b; Cheng et al., 2018; Jiang et al., 2019; Li et al., 2019; Zhang et al., 2019; Hu et al., 2019; Chen et al., 2020; Qu et al., 2020; Meng et al., 2020; Gao, 2021; Chen, 2022; Shen et al., 2022; Wang, 2022; Zhou et al., 2022; Qiao, 2023; Zhang Y. et al., 2024; Liu et al., 2025; Sun, 2025).

### Characteristics of the included patients

The characteristics of the participants and the included studies are shown in Table 2. The final quantitative analysis included 40 RCTs with 3,083 VCI patients with diverse treatment strategies. In these studies, the patients were diagnosed with: VD (75%), VCIND (10%), PSCI (10%), VCI (5%), or MID (2.5%). The proportion of male patients varied from 40% to 73%, with a mean age of 58.0 ± 4.2 to 71.8 ± 3.9 years old. The included studies used similar inclusion and exclusion criteria. Patients treated with herbal medicines were excluded. All retrieved studies were parallel RCTs that compared the benefits of acupuncture therapy on cognitive status and ADL. The patients received different treatments for 4–12 weeks. Details of the interventions are listed in Table 3.

**TABLE 2:** Summary of the retrieved studies.

References	Diagnosis	Age (mean ± SD)	Male, *n* (%)	Sample size (A/C)	Acupuncture arm	Comparison arm	Pharmacotherapy	Outcomes	Endpoint (week)	Ref.
Mo et al., 2000b	VD	67.26 ± 6.34	21 (67.7%)	31/62	EA+MA+SC	P+SC	Ergoloid	HDS, BI, FAQ	6	Mo et al., 2000b
Mo et al., 2000a,b	VD	65.38 ± 5.76	19 (63.3%)	30/60	EA+MA+SC	P+SC	Ergoloid	HDS, BI, FAQ	6	Mo et al., 2000a,b
Niu, 2007	VD	67.08 ± 6.12	12 (40%)	30/60	SA+SC	P+SC	Duxil	HDS	10	Niu, 2007
Chen et al., 2009	VD	68.45 ± 2.35	16 (50%)	32/63	MA+SA+SC	P+SC	GM1	HDS	4	Chen et al., 2009
Meng et al., 2009	VD	66.94 ± 4.88	18 (60%)	30/60	MA+SA+SC	P+SC	Nimodipine	MMSE, HDS	4	Meng et al., 2009
Zhao et al., 2009	VD	62.5 ± 3.4	No data available	No data available	EA+SA+SC	P+SC	Nimodipine	MMSE	6	Zhao et al., 2009
		62.5 ± 3.4	No data available	No data available	EA+SA+P+SC					
Yin et al., 2011	VD	62.67 ± 5.10	18 (60%)	30/60	EA+SA+SC	P+SC	Nimodipine	MMSE	12	Yin et al., 2011
Li P. et al., 2012	VD	65 ± 5	29 (44.3%)	48/98	MA+SA+SC	P+SC	Nimodipine	MMSE, HDS, BI	4	Li P. et al., 2012
Li S. et al., 2012	VD	63 ± 10	21 (55.3%)	38/77	SA+SC	P+SC	Citicoline	MMSE, BI	4	Li S. et al., 2012
Li W. et al., 2012	VD	68.29 ± 8.22	30 (62.5%)	48/94	EA+SA+P+SC	P+SC	Nimodipine	MMSE, HDS	12	Li W. et al., 2012
Teng and Lai, 2012	VD	66 ± 6	16 (59.3%)	27/80	SA+SC	P+SC	Duxil	MMSE, BI	4	Teng and Lai, 2012
		68 ± 6	14 (56%)	25/80	MA+SC					
Lin et al., 2012	MID	62 ± 5	11 (55%)	20/40	EA+SA+SC	P+SC	Oxiracetam	MMSE, ADLS	4	Lin et al., 2012
Zhao, 2013	VD	63.8	22 (73.3%)	30/60	MA+SA+SC	P+SC	Duxil	MMSE, ADLS	8	Zhao, 2013
Cao et al., 2014	VD	65 ± 4	25 (62.5%)	40/80	SA+SC	P+SC	Nicergoline	HDS	4	Cao et al., 2014
Li and Jiao, 2014	VD	66.83 ± 7.36	26 (52%)	50/100	MA+SC	P+SC	Oxiracetam	MMSE, ADAS-cog, ADLS	12	Li and Jiao, 2014
Li et al., 2014	VD	63 ± 9	16 (51.6%)	31/60	AA+SA+SC	P+SC	Aniracetam	MMSE, ADL	6	Li et al., 2014
		64 ± 8	14 (50%)	28/60	MA+SC					
Cui et al., 2015	VD	67 ± 5	14 (46.7%)	30/60	MA+SA+P+SC	P+SC	Nimodipine	MMSE, ADLS	4	Cui et al., 2015
Luo et al., 2015	VD	71.75 ± 3. 87	15 (50%)	30/60	MA+SC	P+SC	Donepezil	MMSE	8	Luo et al., 2015
Zhang et al., 2015	VD	68 ± 5	32 (53.3%)	20/40	MA+SA+SC	P+SC	Nimodipine	MMSE, ADLS	4	Zhang et al., 2015
Yang W. H. et al., 2016	VD	67.61 ± 10.54	38 (63.3%)	60/120	MA+P+SC	P+SC	Nicergoline	ADAS-cog, ADLS	12	Yang W. H. et al., 2016
Tan et al., 2017	VD	66.73 ± 3.12	17 (56.7%)	30/60	MA+SA+SC	P+SC	Nimodipine	MMSE	4	Tan et al., 2017
Wang, 2017b	VD	65.6 ± 6.8	22 (51.2%)	43/129	MA+SC	SC	**–**	MMSE, BI	12	Wang, 2017b
		66.2 ± 6.2	20 (46.5%)	43/129	MA+SA+SC					
Wang, 2017a,b	VD	64.7 ± 7.3	21 (52.5%)	21/40	MA+SC	SC	**–**	MMSE, BI	5	Wang, 2017a,b
		69.3 ± 8.2	23 (57.5%)	23/40	MA+SA+SC					
Cheng et al., 2018	VCI	65.96 ± 11.83	12 (48%)	25/51	MA+SA+SC	P+SC	Donepezil	MMSE	12	Cheng et al., 2018
Hu et al., 2019	VD	68 ± 9	24 (54.5%)	44/88	SA+P+SC	P+SC	Oxiracetam	MMSE, BI	8	Hu et al., 2019
Jiang et al., 2019	VCIND	58 ± 4.2	17 (56.7%)	30/60	EA+SA+CR+SC	CR+SC	**–**	MMSE, MoCA, BI	8	Jiang et al., 2019
Li et al., 2019	PSCI	66.9 ± 5.9	18 (45%)	40/80	MA+SA+P+SC	P+SC	Donepezil	MMSE, MoCA, BI	6	Li et al., 2019
Zhang et al., 2019	VCIND	58.63 ± 8.24	16 (43.2%)	41/82	MA+SC	P+SC	Donepezil	MoCA, BI	8	Zhang et al., 2019
Chen et al., 2020	PSCI	65.3 ± 6.8	16 (57.1%)	28/56	SA+P+SC	P+SC	Donepezil	MoCA, ADLS	4	Chen et al., 2020
Meng et al., 2020	PSCI	65.2 ± 3.7	33 (54.1%)	61/122	MA+SA+P+SC	P+SC	Donepezil	MMSE, MoCA, BI	4	Meng et al., 2020
Qu et al., 2020	VCIND	64.58 ± 2.62	17 (56.7%)	30/60	MA+P+SC	P+SC	Oxiracetam	MMSE, MoCA, BI	4	Qu et al., 2020
Gao, 2021	PSCI	59.35 ± 6.24	19 (50%)	38/75	MA+CR+SC	CR+SC	**–**	MMSE	8	Gao, 2021
Zhou et al., 2022	VD	64.24 ± 7.16	17 (56.7%)	30/60	MA+P+SC	P+SC	Donepezil	MMSE, ADLS	8	Zhou et al., 2022
Shen et al., 2022	VCI	81.83 ± 8.70	12 (40%)	30/60	SA+CR+SC	CR+SC	**–**	MMSE, ADLS	12	Shen et al., 2022
Wang, 2022	VD	69 ± 4	19 (32.2%)	59/116	AA+MA+CR+SC	CR+SC	**–**	MMSE, MoCA, HDS	6	Wang, 2022
Chen, 2022	VD	71.53 ± 17.47	21 (60%)	35/60	MA+SA+SC	MA+SC	**–**	MMSE, ADLS	4	Chen, 2022
Qiao, 2023	VD	69.13 ± 6.31	25 (62.5%)	40/80	MA+SC	SC	**–**	MMSE, MoCA	3	Qiao, 2023
Zhang et al., 2024	VCIND	59.41 ± 6.79	31 (57.4%)	53/105	MA+SC	SC	Piracetam	MMSE, MoCA	8	Zhang et al., 2024
Liu et al., 2025	VD	69.04 ± 3.30	43 (44.8%)	96/114	MA+SA+P+CR+SC	P+CR+SC	Donepezil	MMSE, ADL	12	Liu et al., 2025
Sun, 2025	VD	57.83 ± 6.03	13 (43.3%)	30/60	EA+MA+P+SC	P+SC	Donepezil	MMSE, MoCA, ADL	4	Sun, 2025

A, acupuncture arm; AA, auricular acupuncture; ADAS-cog, Alzheimer’s Disease Assessment Scale-cognitive subscale; ADLS, activities of daily living scale; BI, Barthel Index; CR, cognitive rehabilitation; EA, electroacupuncture; FAQ, Functional Activities Questionnaire; HDS, Hasegama’s Dementia Scale; MA, manual acupuncture; MID, multi-infarct dementia; MMSE, Mini-Mental Status Examination; MoCA, Montreal Cognitive Assessment; PSCI, poststroke cognitive impairment; SA, scalp acupuncture; SC, standard care; SD, standard deviation; P, pharmacotherapy; VCI, vascular cognitive impairment; VCIND, vascular cognitive impairment with non-dementia; VD, vascular dementia.

**TABLE 3:** Summary of the acupuncture treatment details.

References	Acupuncture arm	Acupuncture treatment variables					Acupuncture point formula	Ref.
		Needle retention time (min.)	Weekly treatment frequency (sessions/ week)	Treatment course (week)	Total number of treatment sessions	Cumulative treatment duration (min.)		
Mo et al., 2000b	EA+MA+SC	30	5	6	30	900	EX-HN1, GB20, PC6, GB13, GB39, GV16, GV14, BL18, BL23, ST36, SP6, KI3, LR3, LR2, GB43, HT7, ST40, SP10	Mo et al., 2000b
Mo et al., 2000a,b	EA+MA+SC	30	5	6	30	900	GB20, EX-HN1, PC6, GB13, GB39, GV16, GV14, BL18, BL23, ST36, SP6, KI3, LR3, LR2, GB43, HT7, ST40, SP10, LI15, LI11, LI4, TE5, GB30, GB34	Mo et al., 2000a,b
Niu, 2007	SA+SC	30	7	10	70	2,100	GV24, GB15, GB13, ST1, GB8, GB7, EX-HN1, GV20, BL9	Niu, 2007
Chen et al., 2009	MA+SA+SC	30	6	4	24	720	PC6, GV26, SP6, GV20, EX-HN1, BL18, BL23, CV6, BL17, ST40, CV12, BL40	Chen et al., 2009
Meng et al., 2009	MA+SA+SC	30	6	4	24	720	GV20, GV18, GV17, GB13, GB20, ST36	Meng et al., 2009
Zhao et al., 2009	EA+SA+SC	30	5	6	30	900	EX-HN1, GV20, GV24, GB20	Zhao et al., 2009
	EA+SA+P+SC	30	5	6	30	900	EX-HN1, GV20, GV24, GB20	
Yin et al., 2011	EA+SA+SC	30	6	12	72	2,160	EX-HN1, GV24, GV20	Yin et al., 2011
Li P. et al., 2012	MA+SA+SC	45	7	4	28	1,260	GV20, GV16, GV15, GV24, GV26, GV14, BL67, GV3, GV1, LR3, KI3, ST40, ST36, GB20, GB34, LI11, SP6, SP10, PC6, CV17, HT7, EX-HN3	Li P. et al., 2012
Li S. et al., 2012	SA+SC	30	6	4	24	720	Middle line of forehead, anterior oblique line of vertex-temporal, middle line vertex, posterior occipital line, lateral line 1 of occiput	Li S. et al., 2012
Li W. et al., 2012	EA+SA+P+SC	40	6	12	72	2,880	GV20, GV24, BL4, EX-HN1, GB20, PC6, LI4, ST36, SP6, KI3, KI6, ST40, CV12, BL17, SP10, CV6, CV4	Li W. et al., 2012
Teng and Lai, 2012	SA+SC	30	5	4	20	600	Emotion area, GB20, blood supply point, EX-HN14, GV16, GV20, EX-HN1	Teng and Lai, 2012
	MA+SC	30	5	4	20	600	GV20, EX-HN1, KI3, LR3, GB39, ST36	
Lin et al., 2012	EA+SA+SC	30	7	4	28	840	GV24, ST1, GV20, EX-HN1, PC6, SP6	Lin et al., 2012
Zhao, 2013	MA+SA+SC	30	5	8	40	1,200	GV20, EX-HN1, GV24, GB8, ST1, GB20, KI3, SP6, CV4, CV6, ST40, GV26, PC6, SP10, HT7, LR3	Zhao, 2013
Cao et al., 2014	SA+SC	30	12	4	48	1,440	GV26, GV20, EX-HN1, GB20, GB12, BL10, PC6, ST40, SP6	Cao et al., 2014
Li and Jiao, 2014	MA+SC	40	6	12	72	2,880	GV15, PC8, SP6, KI1, KI3, CV12, GB30, ST36, LI4	Li and Jiao, 2014
Li et al., 2014	AA+SA+SC	30	6	6	36	1,080	SA: middle line of forehead, middle line vertex, anterior temporal line, posterior temporal line; AA: brainstem point, kidney point, shenmen, occipital point	Li et al., 2014
	MA+SC	30	6	6	36	1,080	GV20, EX-HN1, GB20, GV16, LI4, GV26, PC6, ST36, LR3	
Cui et al., 2015	MA+SA+P+SC	30	7	4	28	840	GV20, GV16, GV15, GV24, GV26, GV14, BL67, GV3, BL18, BL23, PC6, BL17, ST40, CV12, ST36	Cui et al., 2015
Luo et al., 2015	MA+SC	30	5	8	40	1,200	CV17, CV12, CV6, SP10, ST36, TE5	Luo et al., 2015
Zhang et al., 2015	MA+SA+SC	40	6	4	24	960	GV20, GV16, GV15, GV24, GV26, GV14, BL67, GV3, GV1, BL18, BL23, BL20, CV6, BL17, PC6, ST40, CV12, ST36	Zhang et al., 2015
Yang W. H. et al., 2016	MA+P+SC	30	7	12	84	2,520	CV6, CV12, CV17, SP10, ST36, TE5	Yang W. H. et al., 2016
Tan et al., 2017	MA+SA+SC	30	7	4	28	840	GV20, GV24, GV26, PC6, PC7, PC8, GV14, GV16, CV17, LI4, ST36, LR3	Tan et al., 2017
Wang, 2017b	MA+SC	20	7	12	84	1,680	GB20, blood-supply acupoint, KI3, GV20, GV14, GV4, SP10	Wang, 2017b
	MA+SA+SC	20	7	12	84	1,680	MA: GB20, blood-supply acupoint, KI3, GV20, GV14, GV4, SP10; SA: GB13, GV24, EX-HN1, HT7, GV11	
Wang, 2017a,b	MA+SC	20	7	5	35	700	GV26, GV14, ST36, GB13, BL40	Wang, 2017a,b
	MA+SA+SC	20	7	5	35	700	MA: GV26, GV14, ST36, GB13, BL40; SA: GV20, EX-HN1	
Cheng et al., 2018	MA+SA+SC	30	6	12	72	2,160	GV24, GV20, GV16, GV14	Cheng et al., 2018
Hu et al., 2019	SA+P+SC	30	6	8	48	1440	Parietal region, anterior parietal region, forehead region, occipital region, lower occipital region, neck region, temporal region	Hu et al., 2019
Jiang et al., 2019	EA+SA+CR+SC	45	5	8	40	1,800	GV20, GV24	Jiang et al., 2019
Li et al., 2019	MA+SA+P+SC	30	5	6	30	900	GV24, GV20, EX-HN1, CV6, CV4, LI4, ST36, LR3	Li et al., 2019
Zhang et al., 2019	MA+SC	20	12	8	96	1,920	GV26, PC6, SP6, EX-HN1, GB39, KI3	Zhang et al., 2019
Chen et al., 2020	SA+P+SC	30	12	4	48	1,440	Parietal region, frontal region, temporal region, occipital region, suboccipital region, anterior parietal region	Chen et al., 2020
Meng et al., 2020	MA+SA+P+SC	30	6	4	24	720	GV20, GV24, GV26, EX-HN3, GV16, KI3, LR3, ST40, PC6, SP6	Meng et al., 2020
Qu et al., 2020	MA+P+SC	30	12	4	48	1,440	GV26, PC6, SP6, GB39, KI3, EX-HN1	Qu et al., 2020
Gao, 2021	MA+CR+SC	30	5	8	40	1,200	BL23, CV4, SP10, LI4, KI3, GB13, GV24, GV20, ST36, SP6, EX-HN1, HT7, GB39	Gao, 2021
Zhou et al., 2022	MA+P+SC	30	6	8	48	1,440	HT7, BL18, PC6, PC4, CV17, CV14, BL15, BL14	Zhou et al., 2022
Shen et al., 2022	SA+CR+SC	60	3	12	36	2,160	GV24, GB8, EX-HN5, GB20, GV16, GV20	Shen et al., 2022
Wang, 2022	AA+MA+CR+SC	20	7	6	42	840	MA+SA: GV20, EX-HN1, GV24, KI1, BL23, KD3, BL18, SP6, CV4, PC1, LU1, ST40, ST36, CV12, BL20, HT3, SJ5, SP10, LI4; AA: yuanzhong, shenmen, occipital point, Diaphragm point, forehead point, subcortical area point	Wang, 2022
Chen, 2022	MA+SA+SC	30	6	4	24	720	MA: LI15, LI11, LI4, SJ5, GB30, GB34, ST36, SP6; SA: midline of the vertex, midline of the forehead, anterior temporal lin, posterior temporal line	Chen, 2022
Qiao, 2023	MA+SC	30	7	3	21	630	KI1, KI3, KI4, KI5, KI6, GV20, GV24, EX-HN1, GV26, Cervical Jiaji, PC6, SP6;	Qiao, 2023
Zhang et al., 2024	MA+SC	30	6	8	48	1,440	GV24, KI3, HT7, ST36, GV20	Zhang et al., 2024
Liu et al., 2025	MA+SA+P+ CR+SC	30	5	12	60	1,800	GV20, EX-HN1, GB20, KI3, ST36, ST40, SP6	Liu et al., 2025
Sun, 2025	EA+MA+P+SC	30	5	4	20	600	KI1, PC6	Sun, 2025

AA, auricular acupuncture; CR, cognitive rehabilitation; EA, electroacupuncture; MA, manual acupuncture; P, pharmacotherapy; SA, scalp acupuncture, SC, standard care.

### Primary outcome: cognitive status

After conducting network meta-analysis with the 40 included RCTs, we observed that five studies could not be analyzed in the connected network structure (Jiang et al., 2019; Gao, 2021; Shen et al., 2022; Wang, 2022; Liu et al., 2025). Hence we excluded the five studies for the following analysis (Figure 1).

The initial network meta-analysis included 35 RCTs involving 2,628 VCI patients and 13 different interventions for cognitive status outcomes. The analysis yielded a stable and consistent network structure (Mo et al., 2000a,b; Niu, 2007; Chen et al., 2009; Meng et al., 2009; Zhao et al., 2009; Yin et al., 2011; Li P. et al., 2012; Li S. et al., 2012; Li W. et al., 2012; Lin et al., 2012; Teng and Lai, 2012; Zhao, 2013; Cao et al., 2014; Li and Jiao, 2014; Li et al., 2014; Cui et al., 2015; Luo et al., 2015; Zhang et al., 2015; Yang W. H. et al., 2016; Tan et al., 2017; Wang, 2017a,b; Cheng et al., 2018; Li et al., 2019; Zhang et al., 2019; Hu et al., 2019; Chen et al., 2020; Qu et al., 2020; Meng et al., 2020; Chen, 2022; Zhou et al., 2022; Qiao, 2023; Zhang Y. et al., 2024; Liu et al., 2025; Sun, 2025). The initial network meta-analysis results are presented in Supplementary Figure 1. The network plot in Supplementary Figure 1A shows that the P+SC group had the largest number of studies, and the comparison between the MA+SA+SC and P+SC groups revealed the largest number of trials. The forest plot of the network meta-analysis (Supplementary Figure 1B) indicated that among all treatments, SA+P+SC [standardized mean difference (SMD): 2.14; 95% confidence interval (CI): 1.04–3.24] was the most effective intervention compared to SC in improving cognitive status. The treatment that ranked second-highest was MA+SA+SC (SMD: 1.84; 95% CI: 1.18–2.50) and had contributed to cognitive status improvements compared to SC. All in all, any type of acupuncture treatment (AA, EA, MA, SA) combined with P or SC was significantly more effective than SC alone in improving cognitive status. Statistical heterogeneity was observed among the included studies, with an I^2^ of 82.5% (95% CI: 75.7–87.4). According to the league table (Supplementary Table 4) and the P-scores (Supplementary Figure 1C), SA+P+SC was identified as the most effective treatment for improving cognitive status.

### Inconsistency assessment

We assessed the consistency assumption of the initial network meta-analysis using the node-splitting model, which compares direct and indirect estimates for each pairwise comparison. No significant inconsistency was observed in any comparison (all *p* > 0.05). Detailed results of the node-splitting inconsistency analysis are presented in Supplementary Table 5. These results indicate that the initial network meta-analysis is consistent.

### Publication bias

To assess the presence of publication bias, a funnel plot was constructed based on the standardized mean differences (SMDs) across studies (Supplementary Figure 1D). Visual inspection of the plot revealed a marked asymmetry, with several studies clustering in the lower right quadrant, indicating disproportionately large effect sizes in studies with higher standard errors. Specifically, studies labeled as #12, 21, 24, 31, and 37 (Lin et al., 2012; Tan et al., 2017; Cheng et al., 2018; Qu et al., 2020; Qiao, 2023) were located in this region, characterized by small sample sizes and unusually high treatment effects. This distribution suggests a potential selective publication of positive results. Meanwhile, Egger’s test was statistically significant (*p* = 0.0011), supporting the presence of small-study effects and publication bias. These findings revealed that studies with favorable outcomes may be more likely to be published, leading to an overestimation of the overall treatment effect in the network meta-analysis.

### Sensitivity analysis

Five studies (#12, 21, 24, 31, and 37) (Lin et al., 2012; Tan et al., 2017; Cheng et al., 2018; Qu et al., 2020; Qiao, 2023) were identified as having both small sample sizes and unusually large effect sizes. These studies were classified as high-risk for publication bias and subsequently excluded in a sensitivity analysis to assess the robustness of the network meta-analysis results. A publication bias-adjusted network meta-analysis was conducted using the remaining studies to determine whether the exclusion of high-risk publications substantially altered treatment effect estimates or treatment rankings.

### Publication bias-adjusted network meta-analysis

The publication bias-adjusted network meta-analysis included studies with cognitive status outcome, for which 13 interventions were included for comparison in 30 RCTs with 2337 VCI patients. The publication bias-adjusted network meta-analysis results are presented in Supplementary Figure 2. The network plot is shown in Supplementary Figure 2A. The forest plot of the publication bias-adjusted network meta-analysis (Supplementary Figure 2B) indicated that among all treatments, SA+P+SC (SMD: 2.14; 95% CI: 1.29–2.99) was the most effective intervention compared to SC in improving cognitive status. All in all, any type of acupuncture treatment (AA, EA, MA, SA) along with conventional treatment was significantly better in improving cognitive status compared to SC. Statistical heterogeneity was observed among the included studies, with an I^2^ of 82.5% (95% CI: 75.7–87.4). According to the league table (Supplementary Table 6) and the P-scores (Supplementary Figure 2C), SA+P+SC was identified as the most effective treatment for improving cognitive status, which is consistent with the findings from the initial network meta-analysis. A funnel plot (Supplementary Figure 2D) was generated to assess the potential for small-study effects or publication bias. The plot appears symmetrical, and the Egger’s test for funnel plot asymmetry was not statistically significant (*p* = 0.4151), suggesting no evidence of publication bias. Most data points were distributed within the pseudo 95% confidence region, and no clear asymmetry was observed.

However, in the publication bias-adjusted network meta-analysis, three comparisons (MA+SA+SC vs MA+SC, MA+SA+SC vs P+SC, and MA+SC vs P+SC) demonstrated statistically significant inconsistency in the node-splitting analysis (*p* < 0.05) (Supplementary Table 7). This suggests that for these specific comparisons, direct and indirect evidence may not be fully coherent, potentially due to residual heterogeneity or sparse evidence. Hence, a post-hoc sensitivity analysis was then conducted by simultaneously excluding four studies (Chen et al., 2009; Meng et al., 2009; Teng and Lai, 2012; Li and Jiao, 2014) that contributed most substantially to the comparisons exhibiting statistical inconsistency in the node-splitting analysis. This approach aimed to evaluate whether the observed inconsistencies were driven by conflicting direct and indirect evidence from these specific studies. We then conducted the consistency-verified network meta-analysis.

### Consistency-verified network meta-analysis

**FIGURE 2:**
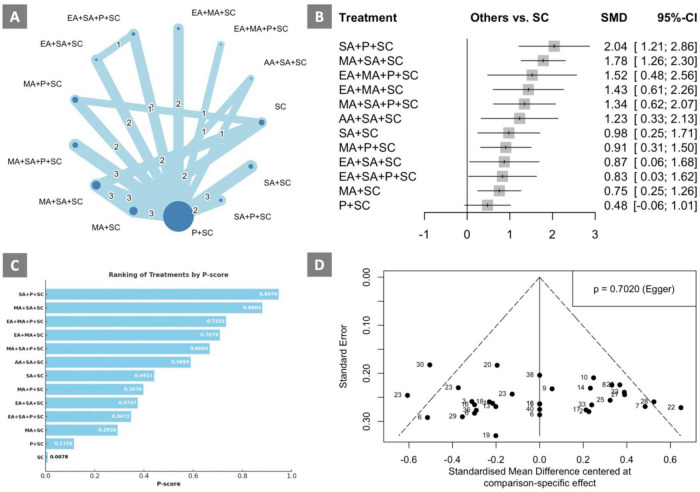
Consistency-verified network meta-analysis results of treatments with cognition status outcomes **(A)** Network plot; **(B)** Forest plot; **(C)** P-scores; **(D)** Funnel plot. Network plot: the size of the nodes corresponds to the number of studies for each treatment. The lines between nodes represent a direct comparison of the trials, and the thickness of the line linked between nodes corresponds to the number of trials included. AA, auricular acupuncture; ADL, activities of daily living; CR, cognitive rehabilitation; EA, electroacupuncture; MA, manual acupuncture; P, pharmacotherapy, SA, scalp acupuncture; SC, standard care; SMD, standardized mean difference.

The consistency-verified network meta-analysis included studies with cognitive status outcome, for which 13 interventions were included for comparison in 26 RCTs with 2034 VCI patients (Mo et al., 2000a,b; Niu, 2007; Zhao et al., 2009; Yin et al., 2011; Li P. et al., 2012; Li S. et al., 2012; Li W. et al., 2012; Zhao, 2013; Cao et al., 2014; Li and Jiao, 2014; Cui et al., 2015; Luo et al., 2015; Zhang et al., 2015; Yang W. H. et al., 2016; Wang, 2017a,b; Li et al., 2019; Zhang et al., 2019; Hu et al., 2019; Chen et al., 2020; Meng et al., 2020; Chen, 2022; Zhou et al., 2022; Zhang Y. et al., 2024; Sun, 2025). The consistency-verified network meta-analysis results are presented in Figure 2. The consistency-verified network plot is shown in Figure 2A. The forest plot of the consistency-verified network meta-analysis (Figure 2B) indicated that among all treatments, SA+P+SC (SMD: 2.04; 95% CI: 1.21–2.86) was the most effective intervention compared to SC in improving cognitive status. All in all, any type of acupuncture treatment (AA, EA, MA, SA) along with conventional treatment was significantly better in improving cognitive status compared to SC. Statistical heterogeneity was observed among the included studies, with an I^2^ of 71.0% (95% CI: 56.2–80.8). According to the league table (Table 4) and the P-scores (Figure 2C), SA+P+SC was identified as the most effective treatment for improving cognitive status, and the treatment that ranked second-highest was MA+SA+SC (SMD: 1.78; 95% CI: 1.26–2.30) and had contributed to cognitive status improvements compared to SC. The results are consistent with the findings from the initial network meta-analysis and the publication bias-adjusted network meta-analysis. As for the inconsistency analysis using node-splitting model, the results showed no significant difference (Supplementary Table 8). To evaluate the presence of publication bias, a comparison-adjusted funnel plot was constructed (Figure 2D). The distribution of studies was approximately symmetrical, with data points evenly scattered around the central vertical line representing the overall effect size. Egger’s test yielded a non-significant result (*p* = 0.7020), indicating no statistical evidence of small-study effects or publication bias.

**TABLE 4:** League table of the consistency-verified network meta-analysis on cognitive status.

SA+P+SC	–	–	–	–	–	–	–	–	–	–	1.56 (0.93, 2.19)	–
0.26 (−0.49, 1.00)	**MA+SA+SC**	–	–	–	–	–	–	–	–	1.28 (0.78, 1.78)	1.04 (0.53, 1.55)	2.00 (1.38, 2.62)
0.52 (−0.58, 1.61)	0.26 (−0.72, 1.24)	**EA+MA+P+SC**	–	–	–	–	–	–	–	–	1.04 (0.15, 1.94)	–
0.60 (−0.28, 1.49)	0.35 (−0.40, 1.09)	0.09 (−1.00, 1.18)	**EA+MA+SC**	–	–	–	–	–	–	–	0.96 (0.33, 1.58)	–
0.70 (−0.10, 1.49)	0.44 (−0.19, 1.07)	0.18 (−0.84, 1.20)	0.09 (−0.70, 0.89)	**MA+SA+P+SC**	–	–	–	–	–	–	0.87 (0.38, 1.35)	–
0.81 (−0.20, 1.81)	0.55 (−0.29, 1.39)	0.29 (−0.90, 1.48)	0.20 (−0.80, 1.21)	0.11 (−0.81, 1.04)	**AA+SA+SC**	–	–	–	–	0.50 (−0.37, 1.38)	0.72 (−0.16, 1.60)	–
1.06 (0.26, 1.86)	0.81 (0.17, 1.44)	0.55 (−0.47, 1.56)	0.46 (−0.34, 1.25)	0.37 (−0.33, 1.06)	0.25 (−0.67, 1.18)	**SA+SC**	–	–	–	–	0.50 (0.01, 0.99)	–
1.13 (0.32, 1.94)	0.88 (0.27, 1.48)	0.62 (−0.41, 1.64)	0.53 (−0.28, 1.34)	0.44 (−0.27, 1.14)	0.32 (−0.60, 1.24)	0.07 (−0.64, 0.78)	**MA+P+SC**	–	–	–	0.45 (-0.14, 1.04)	0.86 (0.05, 1.68)
1.17 (0.29, 2.04)	0.91 (0.18, 1.64)	0.65 (−0.43, 1.73)	0.56 (−0.31, 1.44)	0.47 (−0.31, 1.26)	0.36 (−0.64, 1.35)	0.11 (−0.68, 0.89)	0.04 (−0.76, 0.83)	**EA+SA+SC**	−0.11 (−1.02, 0.79)	–	0.35 (−0.28, 0.99)	–
1.21 (0.35, 2.07)	0.95 (0.24, 1.67)	0.69 (−0.37, 1.76)	0.61 (−0.25, 1.46)	0.51 (−0.25, 1.28)	0.40 (−0.58, 1.38)	0.15 (−0.62, 0.91)	0.08 (−0.70, 0.86)	0.04 (−0.68, 0.77)	**EA+SA+P+SC**	–	0.25 (−0.36, 0.85)	–
1.28 (0.54, 2.03)	1.03 (0.62, 1.44)	0.77 (−0.21, 1.74)	0.68 (−0.06, 1.42)	0.59 (−0.04, 1.22)	0.48 (−0.31, 1.26)	0.22 (−0.41, 0.86)	0.15 (−0.44, 0.75)	0.12 (−0.61, 0.85)	0.07 (−0.63, 0.78)	**MA+SC**	0.52 (0.01, 1.02)	0.61 (0.02, 1.20)
1.56 (0.93, 2.19)	1.30 (0.90, 1.71)	1.04 (0.15, 1.94)	0.96 (0.33, 1.58)	0.87 (0.38, 1.35)	0.75 (−0.03, 1.54)	0.50 (0.01, 0.99)	0.43 (−0.08, 0.94)	0.39 (−0.22, 1.00)	0.35 (−0.24, 0.94)	0.28 (−0.12, 0.67)	**P+SC**	–
2.04 (1.21, 2.86)	1.78 (1.26, 2.30)	1.52 (0.48, 2.56)	1.43 (0.61, 2.26)	1.34 (0.62, 2.07)	1.23 (0.33, 2.13)	0.98 (0.25, 1.71)	0.91 (0.31, 1.50)	0.87 (0.06, 1.68)	0.83 (0.03, 1.62)	0.75 (0.25, 1.26)	0.48 (−0.06, 1.01)	**SC**

Pairwise (upper-right portion) and network (lower-left portion) meta-analysis results are presented as estimate effect sizes for the outcome of changes of cognitive status outcomes in patients with vascular cognitive impairment (VCI). Outcomes are presented as standardized mean difference (SMD) (95% confidence intervals). For the pairwise meta-analyses, SMD of more than 0 indicate that the treatment specified in the row got more beneficial effect than that specified in the column. For the network meta-analysis, SMD of more than 0 indicate that the treatment specified in the column got more beneficial effect than that specified in the row. Gray grids: treatment. Blue grids: treatment in column is significantly more effective than treatment in row. Green grids: treatment in row is significantly more effective than treatment in column. AA, auricular acupuncture; CR, cognitive rehabilitation; EA, electroacupuncture; MA, manual acupuncture; P, pharmacotherapy; SA, scalp acupuncture; SC, standard care.

The final consistency-verified network meta-analysis resolved both publication bias and inconsistency, and its results served as the robust basis for the final interpretation.

### Subgroup analysis

To explore potential sources of heterogeneity in the consistency-verified network meta-analysis (I^2^ = 71.0%, 95% CI: 56.2–80.8), we performed subgroup analyses based on specific diagnostic subtypes of VCI, including VD, MID, VCIND, and PSCI. Among these, only the subgroup of patients diagnosed with VD yielded a stable and consistent network structure. The subgroup network meta-analysis included studies with cognitive status outcome of VD patients, for which 13 interventions were included for comparison in 21 RCTs with 1603 VCI patients (Mo et al., 2000a,b; Niu, 2007; Zhao et al., 2009; Yin et al., 2011; Li P. et al., 2012; Li S. et al., 2012; Li W. et al., 2012; Zhao, 2013; Cao et al., 2014; Li and Jiao, 2014; Li et al., 2014; Cui et al., 2015; Luo et al., 2015; Zhang et al., 2015; Yang W. H. et al., 2016; Wang, 2017a,b; Hu et al., 2019; Chen et al., 2020; Zhou et al., 2022; Sun, 2025). The subgroup network meta-analysis results are presented in Supplementary Figure 3. The subgroup network meta-analysis plot is shown in Supplementary Figure 3A. The forest plot of the subgroup network meta-analysis (Supplementary Figure 3B) indicated that among all treatments, SA+P+SC (SMD: 2.55; 95% CI: 1.58–3.53) was the most effective intervention compared to SC in improving cognitive status of patients with VD. Statistical heterogeneity was observed among the included studies, with an I^2^ of 67.9 % (95% CI: 48.2–80.0). According to the league table (Supplementary Table 9) and the P-scores (Supplementary Figure 3C), SA+P+SC was identified as the most effective treatment for improving cognitive status, which is consistent with the findings from previous consistency-verified network meta-analysis. As for the inconsistency in the node-splitting analysis, the results showed no significant difference (Supplementary Table 10). The scatter of studies appeared generally symmetrical around the central vertical line, and no substantial asymmetry was observed. Egger’s test yielded a *p*-value of 0.5175, indicating no statistically significant evidence of small-study effects or publication bias (Supplementary Figure 3D).

### Meta-regression analysis

To explore potential sources of heterogeneity, meta-regression analyses were conducted using study-level covariates such as participants’ mean age, needle retention time (in minutes), weekly treatment frequency (sessions per week), treatment course (in weeks), total number of treatment sessions, and cumulative treatment duration (total minutes across all sessions).

**FIGURE 3:**
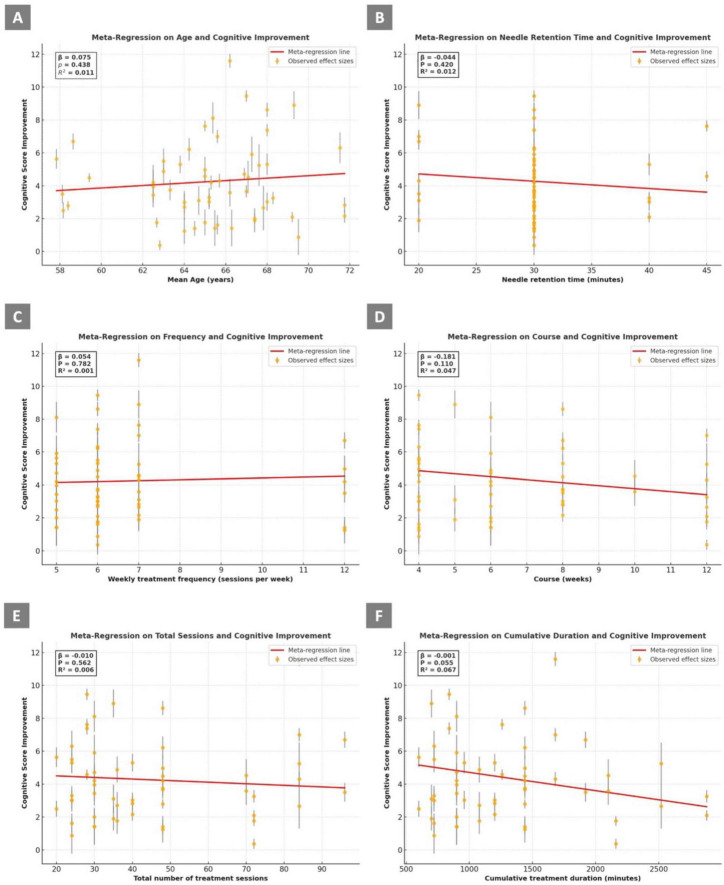
Meta-regression analyses of covariates associated with cognitive improvement **(A)** Mean age (years); **(B)** Needle retention time (minutes); **(C)** Weekly treatment frequency (sessions per week); **(D)** Treatment course (weeks); **(E)** Total number of treatment sessions; **(F)** Cumulative treatment duration (minutes). The results show the relationship between cognitive score improvement and each prespecified study-level covariate. Each dot represents an observed effect size from an individual treatment arm, with vertical bars indicating standard errors. Red lines represent fitted meta-regression lines from weighted least squares (WLS) models. Regression coefficient (β), *p*-value, and R^2^ are presented within each panel.

A meta-regression was conducted to investigate whether the mean age of participants moderated the treatment effect on cognitive outcomes. The analysis indicated no significant association between age and cognitive improvement (β = 0.075, *p* = 0.438, R^2^ = 0.011), suggesting that age did not contribute meaningfully to the variability in treatment effect estimates (Figure 3A).

Meta-regression analysis was performed to evaluate the impact of needle retention time on cognitive outcomes. The analysis showed no significant association (β = –0.044, *p* = 0.420, R^2^ = 0.012), suggesting that needle retention time did not account for the heterogeneity in treatment effects (Figure 3B).

Meta-regression analysis assessing the relationship between weekly acupuncture frequency and cognitive improvement revealed no significant association (β = 0.054, *p* = 0.782, R^2^ = 0.001), indicating that treatment frequency did not explain heterogeneity in outcomes (Figure 3C).

A meta-regression analysis was performed to assess whether the number of treatment weeks (course duration) was associated with cognitive improvement. Although a negative trend was observed (β = −0.181), the association did not reach statistical significance (*p* = 0.110, R^2^ = 0.047), suggesting that course length was not a major source of heterogeneity (Figure 3D).

Meta-regression analysis examining the relationship between the total number of treatment sessions and cognitive improvement revealed no significant association (β = −0.010, *p* = 0.562, R^2^ = 0.006), indicating that the number of sessions did not explain variability in treatment effects (Figure 3E).

Meta-regression analysis showed a marginally significant inverse relationship between cumulative treatment duration and cognitive improvement (β = –0.001, *p* = 0.055, R^2^ = 0.067). This suggests that higher cumulative acupuncture duration may be weakly associated with diminished cognitive benefits, although the result did not reach conventional levels of statistical significance (Figure 3F).

Based on the results of the meta-regression analyses, none of the individual variables (including participants’ mean age, needle retention time, weekly treatment frequency, treatment course, total number of treatment sessions, or cumulative treatment duration) was identified as a major determinant influencing cognitive improvement in patients with VCI.

### Secondary outcome: activity of daily living

Regarding the secondary outcome measurement (ADL), the node-splitting inconsistency test revealed statistically significant inconsistency in multiple treatment comparisons for the ADL outcome, including MA+SA+SC vs MA+SC, MA+SA+SC vs P+SC, MA+SA+SC vs SC, and MA+SC vs SC (all *p* < 0.05). Due to these comparisons involve core treatment nodes and demonstrated substantial differences between direct and indirect estimates, the overall network may lack interpretability and methodological validity. Therefore, a reliable network meta-analysis could not be performed for ADL outcomes based on the available data. Additional high-quality trials with consistent comparator arms are needed to enable robust synthesis of ADL-related outcomes.

### Secondary outcome: severe adverse effects

As a secondary outcome, the occurrence of severe adverse effects associated with the interventions was examined. However, none of the included studies reported any events classified as severe adverse effects. This absence may reflect the generally favorable safety profile of the interventions under investigation, particularly acupuncture-based therapies, which are typically associated with low rates of serious complications. Nevertheless, the lack of severe adverse effects reporting should be interpreted with caution, as it does not necessarily indicate the true absence of such events. Potential underreporting or insufficient safety monitoring in the original studies cannot be ruled out.

### Risk of bias assessment

**FIGURE 4:**
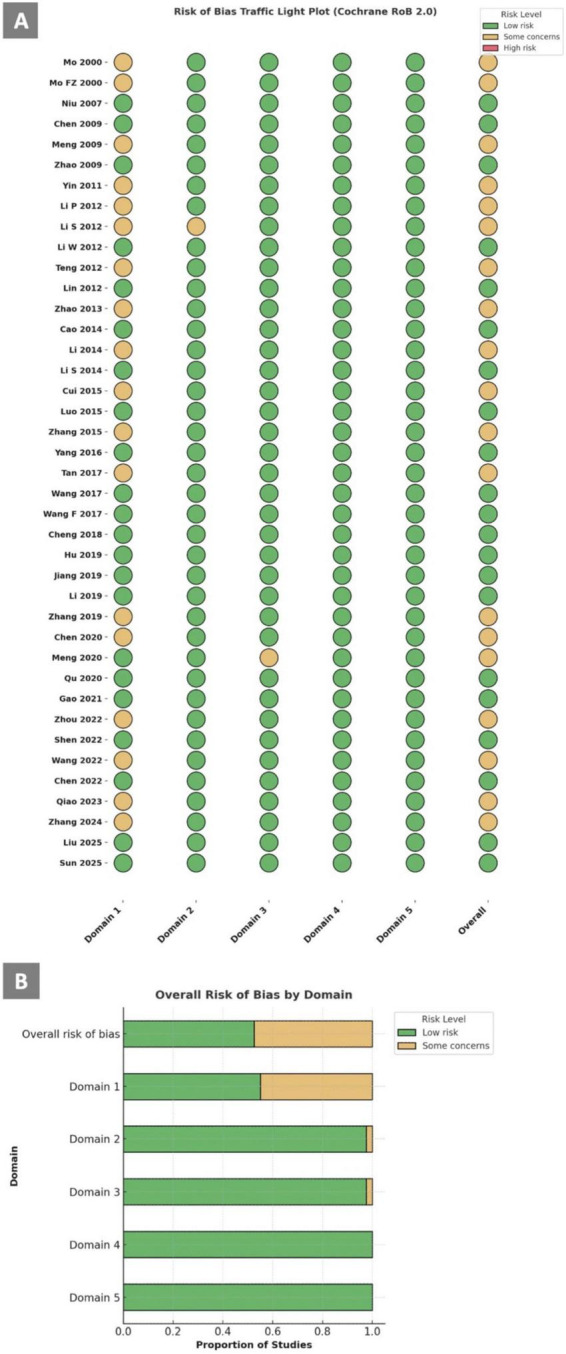
Risk of bias assessment using RoB 2.0 tool **(A)** Traffic light plot; **(B)** Overall risk of bias by domain.

The quality assessment results are presented in Supplementary Table 6. Among the 40 included RCTs, 21 studies were judged to be at “low risk” of bias for all domains, and the other 19 were judged to raise “some concerns” in at least one domain for the result, but not to be at high risk of bias for any domain. The reasons causing risk of bias to the articles were mainly in domain 1, arising from the randomization process which lack specific description of the sequence generation process. The traffic light plot and weighted bar plot are showed in Figure 4. The assessment details are listed in Supplementary Tables 11, 12.

### CINeMA assessment

The certainty of evidence was assessed for each pairwise comparison using the CINeMA framework, which applies the GRADE approach to network meta-analysis. The CINeMA assessment report is presented in Supplementary Table 13.

The comparison SA+P+SC vs SC was rated as having high confidence, as no concerns were identified across all six GRADE domains. Although no direct studies were included in the network for this comparison, the indirect evidence was deemed coherent, precise, and based on studies at low risk of bias, resulting in a robust overall rating. As for the comparison MA+P+SC vs SC was rated as having low confidence. While no concerns were noted regarding indirectness, imprecision, or incoherence, the within-study bias was rated as “some concerns,” and heterogeneity was also identified as an area of concern. These two domains contributed to a downgrading of the overall confidence level. These findings suggest that, while SA+P+SC may have a more reliably estimated effect relative to SC, the evidence for MA+P+SC remains more uncertain due to methodological and variability concerns within the contributing studies.

## Discussion

In the final consistency-verified network meta-analysis, 26 randomized controlled trials involving 2,034 patients with VCI were included. The results indicated that among all treatments, SA+P+SC was the most effective intervention compared to SC in improving cognitive status. Moreover, all types of acupuncture therapies (AA, EA, MA, or SA) combined with P or SC showed significantly greater efficacy in enhancing cognitive function compared to SC alone. No severe adverse effects were reported in any of the included interventions. These findings suggest that acupuncture, particularly SA combined with P or SC, should be considered an effective and safe treatment option for patients with VCI.

In the initial network meta-analysis based on the full dataset, no significant inconsistency was detected via the node-splitting model (all *p* > 0.05), suggesting structural coherence of the network. However, Egger’s test indicated the presence of potential publication bias (*p* < 0.05), raising concerns regarding the overestimation of treatment effects. After excluding five studies with high risk of publication bias, the Egger’s test result improved (non-significant p-value), but inconsistency emerged in several key comparisons, including MA+SA+SC vs. MA+SC, MA+SA+SC vs. P+SC, and MA+SC vs P+SC (all *p* < 0.05) in the publication bias-adjusted network meta-analysis. This suggested that the removal of certain studies disrupted the balance between direct and indirect evidence. To resolve this, four studies were then simultaneously excluded based on their identified contribution to inconsistency. The final consistency-verified network meta-analysis conducted on the refined dataset showed no evidence of publication bias (Egger’s test, *p* > 0.05) and no statistically significant inconsistency across any treatment comparisons (all node-splitting *p* > 0.05). Our sensitivity analysis strategy, which involved stepwise exclusion of studies at high risk of bias, was aligned with recommendations from the Cochrane Handbook and previous methodological research emphasizing the robustness and reliability of the network meta-analysis findings (Donahue et al., 2018; Kiefer et al., 2020; Higgins et al., 2024).

The observed in the final consistency-verified network meta-analysis (I^2^ = 71.0%; 95% CI: 56.2–80.8) may resulted from multiple sources. However, results from subgroup (Supplementary Figure 3C) and meta-regression analyses (Figure 3) indicated that neither the differences in VCI subtype diagnoses nor covariates such as participants’ mean age, needle retention time, treatment frequency, treatment duration, total number of sessions, or cumulative treatment time were major contributors to the observed heterogeneity. The observed heterogeneity may be explained by several factors. First, the diversity of acupuncture interventions across the included studies contributed substantially to the variation. Second, the absence of standardized treatment protocols for VCI patients may have further increased clinical heterogeneity. Additionally, the severity of VCI among participants was not consistently reported, which could have influenced treatment outcomes and added to the variability. Although these sources of heterogeneity exist, they may better reflect real-world clinical settings, where both the presentation of VCI and the application of acupuncture therapies are inherently heterogeneous.

Several recent network meta-analyses have provided valuable insights into the use of non-pharmacological therapies for VCI and VD. Wen et al. (2022) focused on acupuncture modalities and found that combined therapies such as EA+SA+ BA and MB+BA were most effective in improving cognition and daily function among VD patients. Their study highlights the potential of integrated acupuncture techniques. Li et al. (2023) examined interventions in patients with VCIND and concluded that MA combined with traditional Chinese herbal decoction, followed by EA and MA plus piracetam, yielded the greatest cognitive improvements. Their findings emphasize the role of early integrative treatment in delaying disease progression. Yi et al. (2024) expanded the scope to various non-pharmacological therapies for VD and reported that MA with MB and conventional treatment (ACUP_MB_CT) had the highest efficacy for both cognitive and functional outcomes, supporting the utility of multi-modal approaches in dementia care. In contrast to these studies, our analysis included a wider VCI population (PSCI, MID, VCIND and VD) and directly compared four acupuncture types (MA, SA, EA, AA) with P, CR, and SC. We found that SA+P+SC and MA+P+SC were the most effective strategies, and that all acupuncture types combined with P or SC were significantly superior to standard care alone. These findings reinforce the role of acupuncture as a beneficial adjunct in the comprehensive management of VCI.

The minimal clinically important difference (MCID) is an instrument that can target meaningful clinical changes within a group (Jones, 2001; Jones et al., 2016; Alma et al., 2018). In our study, we used standardized mean difference (SMD) to analyze the improvement in cognitive status in the MMSE, HDS, MoCA, and ADAS-cog. According to our study results, SA+P+SC (SMD: 2.04; 95% CI: 1.21–2.86) was the most effective intervention compared to SC in improving cognitive status. However, no study reported the MCID of MMSE, MoCA, HDS or ADAS-cog in VCI patients. Although a previous studies have suggested that a change of 1.4–1.7 points in MMSE may represent the MCID in patients with Alzheimer’s disease (Watt et al., 2021), this threshold may not be directly applicable to individuals with vascular cognitive impairment due to distinct differences in pathophysiology and clinical presentation. Due to the lack of established MCID values for MMSE, MoCA, HDS, and ADAS-cog in VCI patients, we could not confirm whether the observed improvements were clinically meaningful. While SA+P+SC showed the largest effect size, the clinical relevance remains uncertain. Establishing VCI-specific MCID thresholds is essential to better delineate the clinical significance of treatment outcomes.

To further explore the characteristics of the most effective interventions (SA+P+SC and MA+SA+SC), we analyzed the frequency of acupoint selection across the included studies. A total of 65 unique acupoints were extracted from the included studies (Table 3). Among them, GV20 (Baihui) was the most selected point, followed by EX-HN1 (Sishencong), and GV24 (Shenting). Other frequently used points included ST36 (Zusanli), PC6 (Neiguan), KI3 (Taixi), SP6 (Sanyinjiao), GB20 (Fengchi), GV26 (Shuigou), and ST40 (Fenglong). Notably, GV20, EX-HN1, and GV24 are located on the scalp and are commonly used in cognitive-related scalp acupuncture protocols. Others, such as ST36, PC6, and SP6, are manual acupoints associated with general neurological and systemic regulation.

In neuroimaging studies, SA (e.g., GV20 and EX-HN1) and MA (e.g., KI3 and ST36) have been shown to enhance neural activity in the anterior cingulate cortex, hippocampus, and higher-order functional areas within the frontoparietal network, which are critically involved in working memory, attention, and executive decision-making (Zhou and Jin, 2008; Chen et al., 2014; Chung et al., 2019; Wang et al., 2024). Additionally, meta-analyses involving dementia patients have identified these brain regions as among the most functionally impaired and thus important targets for therapeutic stimulation (Cao et al., 2020). Furthermore, manual acupuncture at GB20 has demonstrated effects on cerebral arterial circulation, particularly impacting the basilar artery (Im et al., 2014). Collectively, these findings suggest that acupuncture may confer cognitive benefits through modulation of neural activity in regions associated with attention, cognition, and memory, as well as through improved cerebral blood flow.

These findings suggest a convergence of clinical preference toward specific acupoints for improving cognitive outcomes in patients with VCI. Although these acupoints are commonly used in the included studies and have shown promising effects in the treatment of VCI, there is currently no established standard acupoint combination. Further research is required to determine the optimal combination of acupoints for VCI treatment.

### Limitations

This study had several limitations. First, although 40 RCTs were included, the reported methodological quality was unsatisfactory. The authors rarely specified the details of the method of randomization, the allocation concealment, or blinding of the participants and providers, which led to a certain degree of bias. Second, the included 40 RCTs did not consider the severity of VCI. Therefore, we may obtain a generally recommended treatment for patients with general VCI instead of treatments specified for those with different severities of VCI. Moreover, as for current therapies for VCI patients, no standardized protocols were available in conventional treatment (P and CR), SC or Chinese medicine treatments, which may lead to heterogeneity. Consequently, owing to the diverse treatments in acupuncture, we observed considerable heterogeneity in our study, which was another limitation.

## Conclusion

In the final consistency-verified network meta-analysis of 26 RCTs including 2,034 VCI patients, SA+P+SC was identified as the most effective treatment for improving cognitive status. All acupuncture types (AA, EA, MA, SA) combined with pharmacotherapy or standard care were significantly more effective than standard care alone, with no severe adverse effects reported. These findings suggest that acupuncture, particularly SA combined with P or SC, is a safe and effective adjunctive treatment for vascular cognitive impairment. However, due to the lack of standardized diagnostic criteria, treatment protocols, and clinically established MCID thresholds specific to VCI, the clinical significance of cognitive improvements remains to be fully elucidated. High-quality RCTs with standardized protocols are warranted to further validate these findings.

## Data Availability

The original contributions presented in this study are included in this article/Supplementary material, further inquiries can be directed to the corresponding author.

## Footnotes

1
https://cinema.ispm.unibe.ch/
