# Supplementary material for: Comparisons of acupuncture therapies combining conventional treatment in the management of vascular cognitive impairment: a systematic review and network meta-analysis
Source: Front Aging Neurosci. 2025 Jun 16;17:1559388. doi: 10.3389/fnagi.2025.1559388 (PMC12206881; doi:10.3389/fnagi.2025.1559388)
Supplement: Supplementary file 1 [file Table_1.docx]

**Supplementary Materials**

**Comparisons of Acupuncture Therapies combining Conventional Treatment in the Management of Vascular Cognitive Impairment: A systematic review and network meta-analysis**

Yuan-Ling Liao, Pei-Shan Hsu, Chang-Ti Lee, Li-Jen Su, Yi-Ying Shen, Adam Tsou, Chou-Chin Lan, I-Shiang Tzeng, Guan-Ting Liu, Po-Chun Hsieh*

| **Supplementary Tables** | **Page** |
| --- | --- |
| Supplementary Table 1. PICOS | 3 |
| Supplementary Table 2. Search strategy and results | 4 |
| Supplementary Table 3. List of excluded articles with reasons after full-text evaluation | 5 |
| Supplementary Table 4. League table of the initial network meta-analysis of cognitive status | 7 |
| Supplementary Table 5. Inconsistency test using node-splitting model of the initial network meta-analysis of cognitive status | 8 |
| Supplementary Table 6. League table of the publication bias-adjusted network meta-analysis of cognitive status | 10 |
| Supplementary Table 7. Inconsistency test using node-splitting model of the publication bias-adjusted network meta-analysis of cognitive status | 11 |
| Supplementary Table 8. Inconsistency test using node-splitting model of the consistency-verified network meta-analysis of cognitive status | 13 |
| Supplementary Table 9. League table of the subgroup network meta-analysis of cognitive status in VD patients | 15 |
| Supplementary Table 10. Assessment of inconsistency using the node-splitting model in subgroup analysis | 16 |
| Supplementary Table 11. Risk of bias results assessed by RoB 2 | 18 |
| Supplementary Table 12. Specific reasons for "some concerns" in included studies | 19 |
| Supplementary Table 13. CINeMA assessment report | 20 |

| **Supplementary Figures** | **Page** |
| --- | --- |
| Supplementary Figure 1. Initial network meta-analysis results of the cognitive status | 22 |
| Supplementary Figure 2. Publication bias-adjusted network meta-analysis results of the cognitive status | 23 |
| Supplementary Figure 3. Subgroup network meta-analysis results of cognitive status in VD patients | 24 |

| **Appendix** | **Page** |
| --- | --- |
| PRISMA 2020 Checklist | 25 |

**Supplementary Table 1. PICOS**

| P | Patient diagnosed as vascular cognitive impairment based on established and validated diagnostic definitions (eg. DSM-IV) |
| --- | --- |
| I/C | Acupuncture, manual acupuncture, electroacupuncture, scalp acupuncture, auricular acupuncture, fire needling, warm needling, sham acupuncture, western medicine, usual care, or cognitive rehabilitation. |
| O | Primary outcomes:   1. Cognitive function: MMSE, HDS, MoCA and ADAS-cog. 2. Activities of daily living: ADLS, BI and FAQ.   Secondary outcome:  Risk ratio of the presence of severe adverse effect. |
| S | Randomized controlled trial |

**Supplementary Table 2. Search strategy and results**

| **Search strategy and results in PubMed** | | |  |
| --- | --- | --- | --- |
| **Search** | **Query** | **Results** |  |
| #1 | “Dementia, Vascular”[Mesh] OR “vascular cognitive impairment” | 9,609 |  |
| #2 | “acupuncture” OR “manual acupuncture” OR “scalp acupuncture” OR “electroacupuncture” OR “auricular acupuncture” OR “fire needling” OR “warm needling” | 47,617 |  |
| #3 | #1 AND #2 | 176 |  |
| **Search strategy and results in Embase** | | |  |
| **Search** | **Query** | **Results** |  |
| #1 | 'multiinfarct dementia'/exp | 15,924 |  |
| #2 | ‘vascular cognitive impairment’ | 3,394 |  |
| #3 | ‘acupuncture’ OR ‘manual acupuncture’ OR ‘scalp acupuncture’ OR ‘electroacupuncture’ OR ‘auricular acupuncture’ OR ‘fire needling’ OR ‘warm needling’ | 75,472 |  |
| #4 | (#1 OR #2) AND #3 | 305 |  |
| **Search strategy and results in Cochrane Library** | | |  |
| **Search** | **Query** | **Results** |  |
| #1 | MeSH descriptor: [Dementia, Vascular] explode all trees | 458 |  |
| #2 | “vascular cognitive impairment” | 368 |  |
| #3 | “acupuncture” OR “manual acupuncture” OR “scalp acupuncture” OR “electroacupuncture” OR “auricular acupuncture” OR “fire needling” OR “warm needling” | 24,254 |  |
| #4 | (#1 OR #2) AND #3 | 73 |  |
| **Search strategy and results in China National Knowledge Infrastructure (CNKI)** | | |  |
| **Search** | **Query** | **Results** |  |
| #1 | 血管性认知障碍 + 血管性失智症 + 血管性痴呆 | 13,686 |  |
| #2 | 針刺 + 體針 + 頭皮針 + 電針 + 耳針 + 火針 + 溫針 | 121,914 |  |
| #3 | #1 AND #2 | 646 |  |
| **Search strategy and results in Airiti Library** | | |  |
| **Search** | **Query** | **Results** |  |
| #1 | 血管性認知功能障礙 OR 血管性失智症 OR 血管性癡呆 | 46 |  |
| #2 | 針刺 OR 體針OR 頭皮針 OR 電針 OR 耳針 OR 火針 OR 溫針 | 676 |  |
| #3 | #1 AND #2 | 2 |  |
| **Search strategy and results in WanFang** | | |  |
| **Search** | **Query** | **Results** |  |
| #1 | 血管性认知障碍 OR 血管性失智症 OR 血管性痴呆 | 10,383 |  |
| #2 | 针刺 OR 体针 OR 头皮针 OR 电针 OR 耳针 OR 火针 OR 温针 | 96,212 |  |
| #3 | #1 AND #2 | 496 |  |
| **Search strategy and results in VIP database** | | | |
| **Search** | | **Query** | **Results** |
| #1 | | 血管性认知障碍 OR 血管性失智症 OR 血管性痴呆 | 9,969 |
| #2 | | 针刺 OR 体针 OR 头皮针 OR 电针 OR 耳针 OR 火针 OR 温针 | 87,026 |
| #3 | | #1 AND #2 | 639 |

**Supplementary Table 3. List of excluded articles with reasons after full-text evaluation**

| **Diagnosis not VCI (n=4)** | |
| --- | --- |
| **#** | **Title** |
| 1 | Ji S, Duan J, Hou X, Zhou L, Qin W, Niu H, Luo S, Zhang Y, Chan P, Jin X. The Role of Acupuncture Improving Cognitive Deficits due to Alzheimer's Disease or Vascular Diseases through Regulating Neuroplasticity. *Neural Plast. 2021 Jan 12;2021:8868447.* |
| 2 | Huang L, Yin X, Li W, Cao Y, Chen Y, Lao L, Zhang Z, Mi Y, Xu S. Effects of Acupuncture on Vascular Cognitive Impairment with No Dementia: A Randomized Controlled Trial. *Effects of Acupuncture on Vascular Cognitive Impairment with No Dementia: A Randomized Controlled Trial.* |
| 3 | Tang E.C.-H., Hung C., Lo S.H.-S., Chau J.P.-C., Mok V.C.-T., Lau A.Y.-L. Acupuncture on vascular cognitive impairment associated with cerebral small vessel disease: A systematic review and meta-analysis of randomized and non-randomized controlled trials. *European Journal of Integrative Medicine, 2022-01-01, Volume 49.* |
| 4 | Zhang D., Wei SJ., Wang SR. Clinical Study on the Treatment of Post-Stroke Vascular Dementia (Blood Stasis Obstructing Collaterals Type) with the "Feijing Zouqi" Acupuncture Method. *Journal of Clinical Acupuncture and Moxibustion* 2021; 37(3), 31-35. |
| **Not RCT (n=5)** | |
| **#** | **Title** |
| 1 | Li, Y., Zhao, G., Zhao, J., Xu, W., and Yu, C. Clinical study on acupuncture therapy for vascular dementia. *Inform. On Traditional Chin. Med*. |
| 2 | Zhao, J., Tian, Y., Cheng, Y., Ma, W., Wang, B., and Tong, J. Clinical observation on treating VD with kidney -supplementing & mind -clearing acupuncture method. *Hebei J. Traditional Chin. Med.* |
| 3 | Huang, W., Xu, J., Wang, X., and Sun, S. Study on acupuncture treatment of multi-infarct dementia. *Zhongguo Zhen Jiu.* |
| 4 | Li, X., Zhu SJ., Tang ZS., Luo YF., Fan RJ., Xie GY., Kou YF., Lu Y. Mechanism of electroacupuncture regulating the ligand pathway of tyrosine kinase receptor in improving vascular dementia. Acta Anatomica Sinica, 2023, 54(6): 689-694. |
| 5 | Peng J., Chen X. Electroacupuncture Treatment of 80 Cases of Vascular Dementia Using the "Supplementing Kidney and Unblocking Du, Reviving the Brain and Enhancing Intelligence" Method. *Chinese Acupuncture & Moxibustion* 2022; 42(06), 623-624. |
| **Animal study (n=5)** | |
| **#** | **Title** |
| 1 | Feng, X., Zhang, S., and Huang, C. Influence of acupuncture combined butylphthalide soft capsule on cognitive function and MRI imaging in patients with vascular dementia. *Chin. J. Cardiovasc. Rehab. Med.* |
| 2 | Shi, H., He, F., Dou, W., Huang, B., Li, X., Fan, W., et al. Clinical effect of acupuncture therapy with Jing point for vascular dementia. *J. Fourth Military Med. Univ.* |
| 3 | Liu, Z. B., Niu, W. M., Yang, X. H., and Niu, X. M. Clinical investigation on electroacupuncture treatment of vascular dementia with “Xiusanzhen*”. Zhen Ci Yan Jiu.* |
| 4 | Ding YY., Zhang SX., Liu YL., Yu Y., Yang MG., Liang SX., Liu WL., Tao J. The Effect of Electroacupuncture on Local Consistency of Brain Function in Rats with Vascular Cognitive Impairment. *Chinese Journal of Rehabilitation Theory and Practice* 2022; 28(1), 55-61. |
| 5 | Chen SY., Zhang C.,Gao F.,Zhang XQ.,Yu WT., Wu ZH., Guo F., Dong QB., Zhang HZ. Effects of electroacupuncture with different frequencies on hippocampal neuronal apoptosis and JNK signaling pathway in rats with vascular dementia. *Journal of Acupuncture and Tuina Science* 2022; 20(1):12-21. |
| **Without targeted outcome (n=4)** | |
| **#** | **Title** |
| 1 | Liu, Y., Zhang, H., Chen, G., Wu, W., Hu, J., Wan, W., et al. Therapeutic effects of scalp-acupuncture in patients with vascular dementia induced by cerebral infarction: a randomized controlled trial. *J. Chin. Integr. Med.* |
| 2 | Li WR., Yang QQ., Zhou XH., Wang KP., Li F., Clinical Efficacy of Tongtiao Xinshen Acupuncture Combined with Wenyang Bushen Moxibustion in the Treatment of Vascular Dementia and Its Effects on Cognitive Function. *Chinese Journal of Information on Traditional Chinese Medicine* 2025; (32)1,10. |
| 3 | Zhu Z., Zhang H., Wang Y., Li W., Wu Z., Wu X., and Zhao N. Observation on the Effect of Acupuncture Combined with Donepezil in Treating Vascular Dementia. *Chinese Rural Medicine*, 2024, 31(09): 10-12. |
| 4 | Li HM., Wang XQ., Chen L. Clinical Efficacy Analysis of "Xingnao Kaiqiao" Acupuncture Method in the Treatment of Vascular Dementia. *Diabetes World* 2021; 18(9), 35. |
| **Without targeted treatment (n=5)** | |
| **#** | **Title** |
| 1 | Xiong, J., Zhang, Z., Ma, Y., Li, Z., Zhou, F., Qiao, N., et al. The effect of combined scalp acupuncture and cognitive training in patients with stroke on cognitive and motor functions. *NeuroRehabilitation*. |
| 2 | Li WR., Yang QQ., Zhou XH., Wang KP., Li F., Clinical Efficacy of Tongtiao Xinshen Acupuncture Combined with Wenyang Bushen Moxibustion in the Treatment of Vascular Dementia and Its Effects on Cognitive Function. *Chinese Journal of Information on Traditional Chinese Medicine* 2025; (32)1, 10.19879/j.cnki.1005-5304.202405490 |
| 3 | **Shen ZQ., Cheng HL., Zhang WD. And Bai L.** Clinical Effect of “Tongdu Tiao Shen” Acupuncture Combined with Donepezil in the Treatment of Vascular Mild Cognitive Impairment. China Medical Herald, 2021; 18(26), 68-72. |
| 4 | **Zhang LK., Chen YH., Sun W., et al. Clinical Observation on the Therapeutic Effect of the Xingshen Tongluo Yizhi Acupuncture Method for Vascular Cognitive Impairment. *Journal of Hunan University of Chinese Medicine* 2023, 43(5): 891-896.** |
| 5 | **Huang L, Yin X, Li W, Cao Y, Chen Y, Lao L, Zhang Z, Mi Y, Xu S. Effects of Acupuncture on Vascular Cognitive Impairment with No Dementia: A Randomized Controlled Trial. *J Alzheimers Dis* 2021; 81(4):1391-1401.** |

**Supplementary Table 4. League table of the initial network meta-analysis on cognitive status**

Pairwise (upper-right portion) and network (lower-left portion) meta-analysis results are presented as estimate effect sizes for the outcome of changes of cognitive status outcomes in patients with VCI. Outcomes are presented as standardized mean difference (SMD) (95% confidence intervals). For the pairwise meta-analyses, SMD of more than 0 indicate that the treatment specified in the row got more beneficial effect than that specified in the column. For the network meta-analysis (NMA), SMD of more than 0 indicate that the treatment specified in the column got more beneficial effect than that specified in the row. Grey grids: treatment. Blue grids: treatment in column is significantly more effective than treatment in row. Green grids: treatment in row is significantly more effective than treatment in column. Abbreviations: AA, auricular acupuncture; CR, cognitive rehabilitation; EA, electroacupuncture; MA, manual acupuncture; P, pharmacotherapy; SA, scalp acupuncture; SC, standard care.

| **SA+P+SC** | . | . | . | . | . | . | . | . | . | . | 1.54 (0.67, 2.41) | . |
| --- | --- | --- | --- | --- | --- | --- | --- | --- | --- | --- | --- | --- |
| 0.30 (-0.66, 1.26) | **MA+SA+SC** | . | . | . | . | . | . | . | . | 1.26 (0.56, 1.96) | 1.07 (0.60, 1.54) | 1.98 (1.11, 2.85) |
| 0.51 (-1.00, 2.01) | 0.20 (-1.09, 1.50) | **EA+MA+P+SC** | . | . | . | . | . | . | . | . | 1.03 (-0.20, 2.26) | . |
| 0.60 (-0.47, 1.68) | 0.30 (-0.42, 1.02) | 0.10 (-1.29, 1.48) | **MA+P+SC** | . | . | . | . | . | . | . | 1.17 (0.46, 1.89) | 0.86 (-0.32, 2.03) |
| 0.59 (-0.64, 1.82) | 0.29 (-0.67, 1.24) | 0.08 (-1.42, 1.59) | -0.01 (-1.09, 1.06) | **EA+MA+SC** | . | . | . | . | . | . | 0.95 (0.08, 1.81) | . |
| 0.66 (-0.45, 1.77) | 0.36 (-0.44, 1.16) | 0.16 (-1.25, 1.57) | 0.06 (-0.88, 1.00) | 0.07 (-1.04, 1.18) | **MA+SA+P+SC** | . | . | . | . | . | 0.87 (0.18, 1.56) | . |
| 0.70 (-0.43, 1.82) | 0.39 (-0.43, 1.22) | 0.19 (-1.23, 1.61) | 0.09 (-0.86, 1.05) | 0.11 (-1.02, 1.23) | 0.03 (-0.96, 1.03) | **EA+SA+SC** | . | . | -0.11 (-1.35, 1.13) | . | 0.83 (0.10, 1.57) | . |
| 0.73 (-0.65, 2.12) | 0.43 (-0.70, 1.56) | 0.23 (-1.41, 1.87) | 0.13 (-1.11, 1.37) | 0.14 (-1.24, 1.53) | 0.07 (-1.21, 1.35) | 0.04 (-1.26, 1.33) | **AA+SA+SC** | . | . | 0.50 (-0.72, 1.72) | 0.71 (-0.51, 1.94) | . |
| 0.89 (-0.15, 1.94) | 0.59 (-0.11, 1.29) | 0.39 (-0.97, 1.75) | 0.29 (-0.57, 1.15) | 0.30 (-0.74, 1.35) | 0.23 (-0.68, 1.14) | 0.20 (-0.73, 1.12) | 0.16 (-1.06, 1.38) | **SA+SC** | . | 0.48 (-0.75, 1.72) | 0.64 (0.04, 1.25) | . |
| 1.07 (-0.12, 2.26) | 0.77 (-0.14, 1.68) | 0.57 (-0.91, 2.04) | 0.47 (-0.56, 1.50) | 0.48 (-0.71, 1.67) | 0.41 (-0.66, 1.48) | 0.37 (-0.57, 1.32) | 0.34 (-1.02, 1.69) | 0.18 (-0.83, 1.18) | **EA+SA+P+SC** | . | 0.23 (-0.62, 1.08) | . |
| 1.14 (0.17, 2.12) | 0.84 (0.35, 1.33) | 0.64 (-0.67, 1.94) | 0.54 (-0.19, 1.27) | 0.55 (-0.42, 1.52) | 0.48 (-0.34, 1.30) | 0.45 (-0.39, 1.28) | 0.41 (-0.67, 1.49) | 0.25 (-0.44, 0.94) | 0.07 (-0.85, 1.00) | **MA+SC** | 0.52 (-0.03, 1.06) | 1.06 (0.36, 1.76) |
| 1.54 (0.67, 2.41) | 1.23 (0.83, 1.64) | 1.03 (-0.20, 2.26) | 0.93 (0.30, 1.57) | 0.95 (0.08, 1.81) | 0.87 (0.18, 1.56) | 0.84 (0.13, 1.55) | 0.80 (-0.28, 1.88) | 0.64 (0.06, 1.23) | 0.47 (-0.35, 1.28) | 0.39 (-0.04, 0.83) | **P+SC** | . |
| 2.12 (1.03, 3.20) | 1.81 (1.16, 2.47) | 1.61 (0.22, 3.00) | 1.51 (0.74, 2.29) | 1.53 (0.45, 2.61) | 1.45 (0.51, 2.40) | 1.42 (0.46, 2.38) | 1.38 (0.17, 2.59) | 1.22 (0.37, 2.08) | 1.05 (0.01, 2.09) | 0.97 (0.37, 1.58) | 0.58 (-0.07, 1.23) | **SC** |

**Supplementary Table 5. Assessment of inconsistency using the node-splitting model in initial network meta-analysis**

| **Comparison** | **No. Studies** | **NMA** | **Direct** | **Indirect** | **Difference** | **Diff 95CI lower** | **Diff 95CI upper** | **pValue** |
| --- | --- | --- | --- | --- | --- | --- | --- | --- |
| AA+SA+SC:EA+MA+P+SC | 0 | -0.2321 | NA | -0.2321 | NA | NA | NA | NA |
| AA+SA+SC:EA+MA+SC | 0 | -0.1465 | NA | -0.1465 | NA | NA | NA | NA |
| AA+SA+SC:EA+SA+P+SC | 0 | 0.3401 | NA | 0.3401 | NA | NA | NA | NA |
| AA+SA+SC:EA+SA+SC | 0 | -0.0456 | NA | -0.0456 | NA | NA | NA | NA |
| AA+SA+SC:MA+P+SC | 0 | -0.1381 | NA | -0.1381 | NA | NA | NA | NA |
| AA+SA+SC:MA+SA+P+SC | 0 | -0.0705 | NA | -0.0705 | NA | NA | NA | NA |
| AA+SA+SC:MA+SA+SC | 0 | -0.4393 | NA | -0.4393 | NA | NA | NA | NA |
| AA+SA+SC:MA+SC | 1 | 0.4144 | 0.5042 | 0.0934 | 0.4108 | -2.2469 | 3.0685 | 0.7619 |
| AA+SA+SC:P+SC | 1 | 0.8124 | 0.7226 | 1.1331 | -0.4105 | -3.0663 | 2.2453 | 0.7619 |
| AA+SA+SC:SA+P+SC | 0 | -0.7404 | NA | -0.7404 | NA | NA | NA | NA |
| AA+SA+SC:SA+SC | 0 | 0.1614 | NA | 0.1614 | NA | NA | NA | NA |
| AA+SA+SC:SC | 0 | 1.4020 | NA | 1.4020 | NA | NA | NA | NA |
| EA+MA+P+SC:EA+MA+SC | 0 | 0.0856 | NA | 0.0856 | NA | NA | NA | NA |
| EA+MA+P+SC:EA+SA+P+SC | 0 | 0.5722 | NA | 0.5722 | NA | NA | NA | NA |
| EA+MA+P+SC:EA+SA+SC | 0 | 0.1865 | NA | 0.1865 | NA | NA | NA | NA |
| EA+MA+P+SC:MA+P+SC | 0 | 0.0940 | NA | 0.0940 | NA | NA | NA | NA |
| EA+MA+P+SC:MA+SA+P+SC | 0 | 0.1617 | NA | 0.1617 | NA | NA | NA | NA |
| EA+MA+P+SC:MA+SA+SC | 0 | -0.2072 | NA | -0.2072 | NA | NA | NA | NA |
| EA+MA+P+SC:MA+SC | 0 | 0.6466 | NA | 0.6466 | NA | NA | NA | NA |
| EA+MA+P+SC:P+SC | 1 | 1.0446 | 1.0446 | NA | NA | NA | NA | NA |
| EA+MA+P+SC:SA+P+SC | 0 | -0.5082 | NA | -0.5082 | NA | NA | NA | NA |
| EA+MA+P+SC:SA+SC | 0 | 0.3936 | NA | 0.3936 | NA | NA | NA | NA |
| EA+MA+P+SC:SC | 0 | 1.6341 | NA | 1.6341 | NA | NA | NA | NA |
| EA+MA+SC:EA+SA+P+SC | 0 | 0.4866 | NA | 0.4866 | NA | NA | NA | NA |
| EA+MA+SC:EA+SA+SC | 0 | 0.1009 | NA | 0.1009 | NA | NA | NA | NA |
| EA+MA+SC:MA+P+SC | 0 | 0.0084 | NA | 0.0084 | NA | NA | NA | NA |
| EA+MA+SC:MA+SA+P+SC | 0 | 0.0760 | NA | 0.0760 | NA | NA | NA | NA |
| EA+MA+SC:MA+SA+SC | 0 | -0.2928 | NA | -0.2928 | NA | NA | NA | NA |
| EA+MA+SC:MA+SC | 0 | 0.5609 | NA | 0.5609 | NA | NA | NA | NA |
| EA+MA+SC:P+SC | 2 | 0.9590 | 0.9590 | NA | NA | NA | NA | NA |
| EA+MA+SC:SA+P+SC | 0 | -0.5938 | NA | -0.5938 | NA | NA | NA | NA |
| EA+MA+SC:SA+SC | 0 | 0.3080 | NA | 0.3080 | NA | NA | NA | NA |
| EA+MA+SC:SC | 0 | 1.5485 | NA | 1.5485 | NA | NA | NA | NA |
| EA+SA+P+SC:EA+SA+SC | 1 | -0.3857 | 0.1109 | -1.0839 | 1.1948 | -0.7621 | 3.1516 | 0.2314 |
| EA+SA+P+SC:MA+P+SC | 0 | -0.4782 | NA | -0.4782 | NA | NA | NA | NA |
| EA+SA+P+SC:MA+SA+P+SC | 0 | -0.4106 | NA | -0.4106 | NA | NA | NA | NA |
| EA+SA+P+SC:MA+SA+SC | 0 | -0.7794 | NA | -0.7794 | NA | NA | NA | NA |
| EA+SA+P+SC:MA+SC | 0 | 0.0743 | NA | 0.0743 | NA | NA | NA | NA |
| EA+SA+P+SC:P+SC | 2 | 0.4724 | 0.2323 | 3.0350 | -2.8027 | -5.7733 | 0.1680 | 0.0644 |
| EA+SA+P+SC:SA+P+SC | 0 | -1.0804 | NA | -1.0804 | NA | NA | NA | NA |
| EA+SA+P+SC:SA+SC | 0 | -0.1787 | NA | -0.1787 | NA | NA | NA | NA |
| EA+SA+P+SC:SC | 0 | 1.0619 | NA | 1.0619 | NA | NA | NA | NA |
| EA+SA+SC:MA+P+SC | 0 | -0.0925 | NA | -0.0925 | NA | NA | NA | NA |
| EA+SA+SC:MA+SA+P+SC | 0 | -0.0249 | NA | -0.0249 | NA | NA | NA | NA |
| EA+SA+SC:MA+SA+SC | 0 | -0.3937 | NA | -0.3937 | NA | NA | NA | NA |
| EA+SA+SC:MA+SC | 0 | 0.4600 | NA | 0.4600 | NA | NA | NA | NA |
| EA+SA+SC:P+SC | 3 | 0.8580 | 0.8541 | 0.9330 | -0.0789 | -3.4222 | 3.2644 | 0.9631 |
| EA+SA+SC:SA+P+SC | 0 | -0.6948 | NA | -0.6948 | NA | NA | NA | NA |
| EA+SA+SC:SA+SC | 0 | 0.2070 | NA | 0.2070 | NA | NA | NA | NA |
| EA+SA+SC:SC | 0 | 1.4476 | NA | 1.4476 | NA | NA | NA | NA |
| MA+P+SC:MA+SA+P+SC | 0 | 0.0677 | NA | 0.0677 | NA | NA | NA | NA |
| MA+P+SC:MA+SA+SC | 0 | -0.3012 | NA | -0.3012 | NA | NA | NA | NA |
| MA+P+SC:MA+SC | 0 | 0.5526 | NA | 0.5526 | NA | NA | NA | NA |
| MA+P+SC:P+SC | 3 | 0.9506 | 1.1975 | 0.0141 | 1.1834 | -0.4024 | 2.7692 | 0.1436 |
| MA+P+SC:SA+P+SC | 0 | -0.6022 | NA | -0.6022 | NA | NA | NA | NA |
| MA+P+SC:SA+SC | 0 | 0.2996 | NA | 0.2996 | NA | NA | NA | NA |
| MA+P+SC:SC | 1 | 1.5401 | 0.8641 | 2.0475 | -1.1834 | -2.7692 | 0.4024 | 0.1436 |
| MA+SA+P+SC:MA+SA+SC | 0 | -0.3688 | NA | -0.3688 | NA | NA | NA | NA |
| MA+SA+P+SC:MA+SC | 0 | 0.4849 | NA | 0.4849 | NA | NA | NA | NA |
| MA+SA+P+SC:P+SC | 3 | 0.8829 | 0.8829 | NA | NA | NA | NA | NA |
| MA+SA+P+SC:SA+P+SC | 0 | -0.6699 | NA | -0.6699 | NA | NA | NA | NA |
| MA+SA+P+SC:SA+SC | 0 | 0.2319 | NA | 0.2319 | NA | NA | NA | NA |
| MA+SA+P+SC:SC | 0 | 1.4724 | NA | 1.4724 | NA | NA | NA | NA |
| MA+SA+SC:MA+SC | 3 | 0.8537 | 1.2766 | 0.4485 | 0.8281 | -0.1691 | 1.8253 | 0.1036 |
| MA+SA+SC:P+SC | 7 | 1.2518 | 1.0874 | 1.7550 | -0.6676 | -1.6276 | 0.2924 | 0.1729 |
| MA+SA+SC:SA+P+SC | 0 | -0.3010 | NA | -0.3010 | NA | NA | NA | NA |
| MA+SA+SC:SA+SC | 0 | 0.6007 | NA | 0.6007 | NA | NA | NA | NA |
| MA+SA+SC:SC | 2 | 1.8413 | 2.0116 | 1.6128 | 0.3988 | -0.9412 | 1.7388 | 0.5597 |
| MA+SC:P+SC | 5 | 0.3980 | 0.5230 | 0.1706 | 0.3524 | -0.5746 | 1.2794 | 0.4562 |
| MA+SC:SA+P+SC | 0 | -1.1548 | NA | -1.1548 | NA | NA | NA | NA |
| MA+SC:SA+SC | 1 | -0.2530 | -0.4895 | -0.1451 | -0.3443 | -1.8602 | 1.1715 | 0.6562 |
| MA+SC:SC | 3 | 0.9875 | 1.0712 | 0.7155 | 0.3557 | -1.1045 | 1.8160 | 0.6330 |
| P+SC:SA+P+SC | 2 | -1.5528 | -1.5528 | NA | NA | NA | NA | NA |
| P+SC:SA+SC | 4 | -0.6510 | -0.6535 | -0.6146 | -0.0389 | -2.4957 | 2.4180 | 0.9753 |
| P+SC:SC | 0 | 0.5895 | NA | 0.5895 | NA | NA | NA | NA |
| SA+P+SC:SA+SC | 0 | 0.9018 | NA | 0.9018 | NA | NA | NA | NA |
| SA+P+SC:SC | 0 | 2.1423 | NA | 2.1423 | NA | NA | NA | NA |
| SA+SC:SC | 0 | 1.2405 | NA | 1.2405 | NA | NA | NA | NA |

**Supplementary Table 6. League table of the publication bias-adjusted network meta-analysis on cognitive status**

Pairwise (upper-right portion) and network (lower-left portion) meta-analysis results are presented as estimate effect sizes for the outcome of changes of cognitive status outcomes in patients with VCI. Outcomes are presented as standardized mean difference (SMD) (95% confidence intervals). For the pairwise meta-analyses, SMD of more than 0 indicate that the treatment specified in the row got more beneficial effect than that specified in the column. For the network meta-analysis (NMA), SMD of more than 0 indicate that the treatment specified in the column got more beneficial effect than that specified in the row. Grey grids: treatment. Blue grids: treatment in column is significantly more effective than treatment in row. Green grids: treatment in row is significantly more effective than treatment in column. Abbreviations: AA, auricular acupuncture; CR, cognitive rehabilitation; EA, electroacupuncture; MA, manual acupuncture; P, pharmacotherapy; SA, scalp acupuncture; SC, standard care.

| **SA+P+SC** | . | . | . | . | . | . | . | . | . | . | 1.56 (0.90, 2.22) | . |
| --- | --- | --- | --- | --- | --- | --- | --- | --- | --- | --- | --- | --- |
| 0.49 (-0.26, 1.23) | **MA+SA+SC** | . | . | . | . | . | . | . | . | 1.28 (0.75, 1.81) | 0.79 (0.37, 1.20) | 2.00 (1.35, 2.65) |
| 0.51 (-0.63, 1.66) | 0.03 (-0.97, 1.02) | **EA+MA+P+SC** | . | . | . | . | . | . | . | . | 1.04 (0.11, 1.98) | . |
| 0.60 (-0.33, 1.53) | 0.11 (-0.63, 0.86) | 0.09 (-1.06, 1.23) | **EA+MA+SC** | . | . | . | . | . | . | . | 0.96 (0.30, 1.62) | . |
| 0.69 (-0.15, 1.53) | 0.20 (-0.42, 0.82) | 0.18 (-0.89, 1.24) | 0.09 (-0.75, 0.92) | **MA+SA+P+SC** | . | . | . | . | . | . | 0.87 (0.35, 1.38) | . |
| 0.83 (-0.22, 1.88) | 0.34 (-0.52, 1.20) | 0.31 (-0.93, 1.56) | 0.23 (-0.82, 1.28) | 0.14 (-0.83, 1.10) | **AA+SA+SC** | . | . | . | . | 0.50 (-0.42, 1.43) | 0.72 (-0.20, 1.65) | . |
| 0.93 (0.14, 1.73) | 0.45 (-0.10, 1.00) | 0.42 (-0.61, 1.45) | 0.33 (-0.46, 1.13) | 0.24 (-0.43, 0.92) | 0.11 (-0.81, 1.03) | **SA+SC** | . | . | . | 0.49 (-0.45, 1.43) | 0.65 (0.20, 1.10) | . |
| 1.17 (0.25, 2.09) | 0.68 (-0.05, 1.41) | 0.65 (-0.48, 1.79) | 0.57 (-0.35, 1.49) | 0.48 (-0.35, 1.30) | 0.34 (-0.70, 1.38) | 0.23 (-0.55, 1.01) | **EA+SA+SC** | . | -0.11 (-1.06, 0.84) | . | 0.35 (-0.32, 1.02) | . |
| 1.16 (0.31, 2.02) | 0.68 (0.07, 1.28) | 0.65 (-0.43, 1.73) | 0.56 (-0.29, 1.41) | 0.47 (-0.27, 1.22) | 0.34 (-0.63, 1.30) | 0.23 (-0.46, 0.92) | -0.00 (-0.84, 0.83) | **MA+P+SC** | . | . | 0.45 (-0.17, 1.08) | 0.86 (0.00, 1.73) |
| 1.21 (0.31, 2.11) | 0.72 (0.01, 1.43) | 0.70 (-0.43, 1.82) | 0.61 (-0.30, 1.51) | 0.52 (-0.29, 1.32) | 0.38 (-0.64, 1.41) | 0.27 (-0.48, 1.03) | 0.04 (-0.72, 0.80) | 0.05 (-0.77, 0.87) | **EA+SA+P+SC** | . | 0.24 (-0.40, 0.88) | . |
| 1.32 (0.58, 2.07) | 0.84 (0.45, 1.22) | 0.81 (-0.18, 1.80) | 0.72 (-0.02, 1.46) | 0.63 (0.02, 1.25) | 0.50 (-0.32, 1.31) | 0.39 (-0.13, 0.91) | 0.16 (-0.57, 0.88) | 0.16 (-0.44, 0.76) | 0.12 (-0.59, 0.82) | **MA+SC** | 0.52 (0.11, 0.93) | 0.61 (-0.01, 1.24) |
| 1.56 (0.90, 2.22) | 1.07 (0.73, 1.42) | 1.04 (0.11, 1.98) | 0.96 (0.30, 1.62) | 0.87 (0.35, 1.38) | 0.73 (-0.09, 1.55) | 0.62 (0.18, 1.07) | 0.39 (-0.25, 1.03) | 0.39 (-0.14, 0.93) | 0.35 (-0.27, 0.97) | 0.23 (-0.10, 0.57) | **P+SC** | . |
| 2.14 (1.29, 2.99) | 1.65 (1.12, 2.19) | 1.63 (0.55, 2.71) | 1.54 (0.69, 2.39) | 1.45 (0.71, 2.20) | 1.31 (0.37, 2.26) | 1.21 (0.53, 1.89) | 0.97 (0.14, 1.81) | 0.98 (0.36, 1.59) | 0.93 (0.11, 1.75) | 0.82 (0.29, 1.34) | 0.58 (0.05, 1.12) | **SC** |

**Supplementary Table 7. Assessment of inconsistency using the node-splitting model in publication bias-adjusted network meta-analysis**

| **Comparison** | **No. Studies** | **NMA** | **Direct** | **Indirect** | **Difference** | **Diff 95CI lower** | **Diff 95CI upper** | **pValue** |
| --- | --- | --- | --- | --- | --- | --- | --- | --- |
| AA+SA+SC:EA+MA+P+SC | 0 | -0.3141 | NA | -0.3141 | NA | NA | NA | NA |
| AA+SA+SC:EA+MA+SC | 0 | -0.2264 | NA | -0.2264 | NA | NA | NA | NA |
| AA+SA+SC:EA+SA+P+SC | 0 | 0.3812 | NA | 0.3812 | NA | NA | NA | NA |
| AA+SA+SC:EA+SA+SC | 0 | 0.3395 | NA | 0.3395 | NA | NA | NA | NA |
| AA+SA+SC:MA+P+SC | 0 | 0.3359 | NA | 0.3359 | NA | NA | NA | NA |
| AA+SA+SC:MA+SA+P+SC | 0 | -0.1384 | NA | -0.1384 | NA | NA | NA | NA |
| AA+SA+SC:MA+SA+SC | 0 | -0.3402 | NA | -0.3402 | NA | NA | NA | NA |
| AA+SA+SC:MA+SC | 1 | 0.4962 | 0.5042 | 0.4676 | 0.0366 | -1.9418 | 2.0150 | 0.9711 |
| AA+SA+SC:P+SC | 1 | 0.7305 | 0.7226 | 0.7591 | -0.0365 | -2.0124 | 1.9393 | 0.9711 |
| AA+SA+SC:SA+P+SC | 0 | -0.8285 | NA | -0.8285 | NA | NA | NA | NA |
| AA+SA+SC:SA+SC | 0 | 0.1063 | NA | 0.1063 | NA | NA | NA | NA |
| AA+SA+SC:SC | 0 | 1.3134 | NA | 1.3134 | NA | NA | NA | NA |
| EA+MA+P+SC:EA+MA+SC | 0 | 0.0877 | NA | 0.0877 | NA | NA | NA | NA |
| EA+MA+P+SC:EA+SA+P+SC | 0 | 0.6952 | NA | 0.6952 | NA | NA | NA | NA |
| EA+MA+P+SC:EA+SA+SC | 0 | 0.6535 | NA | 0.6535 | NA | NA | NA | NA |
| EA+MA+P+SC:MA+P+SC | 0 | 0.6500 | NA | 0.6500 | NA | NA | NA | NA |
| EA+MA+P+SC:MA+SA+P+SC | 0 | 0.1757 | NA | 0.1757 | NA | NA | NA | NA |
| EA+MA+P+SC:MA+SA+SC | 0 | -0.0261 | NA | -0.0261 | NA | NA | NA | NA |
| EA+MA+P+SC:MA+SC | 0 | 0.8103 | NA | 0.8103 | NA | NA | NA | NA |
| EA+MA+P+SC:P+SC | 1 | 1.0446 | 1.0446 | NA | NA | NA | NA | NA |
| EA+MA+P+SC:SA+P+SC | 0 | -0.5144 | NA | -0.5144 | NA | NA | NA | NA |
| EA+MA+P+SC:SA+SC | 0 | 0.4203 | NA | 0.4203 | NA | NA | NA | NA |
| EA+MA+P+SC:SC | 0 | 1.6275 | NA | 1.6275 | NA | NA | NA | NA |
| EA+MA+SC:EA+SA+P+SC | 0 | 0.6076 | NA | 0.6076 | NA | NA | NA | NA |
| EA+MA+SC:EA+SA+SC | 0 | 0.5659 | NA | 0.5659 | NA | NA | NA | NA |
| EA+MA+SC:MA+P+SC | 0 | 0.5623 | NA | 0.5623 | NA | NA | NA | NA |
| EA+MA+SC:MA+SA+P+SC | 0 | 0.0880 | NA | 0.0880 | NA | NA | NA | NA |
| EA+MA+SC:MA+SA+SC | 0 | -0.1138 | NA | -0.1138 | NA | NA | NA | NA |
| EA+MA+SC:MA+SC | 0 | 0.7226 | NA | 0.7226 | NA | NA | NA | NA |
| EA+MA+SC:P+SC | 2 | 0.9569 | 0.9569 | NA | NA | NA | NA | NA |
| EA+MA+SC:SA+P+SC | 0 | -0.6021 | NA | -0.6021 | NA | NA | NA | NA |
| EA+MA+SC:SA+SC | 0 | 0.3327 | NA | 0.3327 | NA | NA | NA | NA |
| EA+MA+SC:SC | 0 | 1.5398 | NA | 1.5398 | NA | NA | NA | NA |
| EA+SA+P+SC:EA+SA+SC | 1 | -0.0417 | 0.1109 | -0.3155 | 0.4264 | -1.1594 | 2.0122 | 0.5982 |
| EA+SA+P+SC:MA+P+SC | 0 | -0.0452 | NA | -0.0452 | NA | NA | NA | NA |
| EA+SA+P+SC:MA+SA+P+SC | 0 | -0.5196 | NA | -0.5196 | NA | NA | NA | NA |
| EA+SA+P+SC:MA+SA+SC | 0 | -0.7213 | NA | -0.7213 | NA | NA | NA | NA |
| EA+SA+P+SC:MA+SC | 0 | 0.1150 | NA | 0.1150 | NA | NA | NA | NA |
| EA+SA+P+SC:P+SC | 2 | 0.3494 | 0.2429 | 1.9165 | -1.6736 | -4.2074 | 0.8602 | 0.1955 |
| EA+SA+P+SC:SA+P+SC | 0 | -1.2096 | NA | -1.2096 | NA | NA | NA | NA |
| EA+SA+P+SC:SA+SC | 0 | -0.2749 | NA | -0.2749 | NA | NA | NA | NA |
| EA+SA+P+SC:SC | 0 | 0.9323 | NA | 0.9323 | NA | NA | NA | NA |
| EA+SA+SC:MA+P+SC | 0 | -0.0036 | NA | -0.0036 | NA | NA | NA | NA |
| EA+SA+SC:MA+SA+P+SC | 0 | -0.4779 | NA | -0.4779 | NA | NA | NA | NA |
| EA+SA+SC:MA+SA+SC | 0 | -0.6797 | NA | -0.6797 | NA | NA | NA | NA |
| EA+SA+SC:MA+SC | 0 | 0.1567 | NA | 0.1567 | NA | NA | NA | NA |
| EA+SA+SC:P+SC | 2 | 0.3910 | 0.3491 | 0.9368 | -0.5877 | -3.0815 | 1.9061 | 0.6442 |
| EA+SA+SC:SA+P+SC | 0 | -1.1680 | NA | -1.1680 | NA | NA | NA | NA |
| EA+SA+SC:SA+SC | 0 | -0.2332 | NA | -0.2332 | NA | NA | NA | NA |
| EA+SA+SC:SC | 0 | 0.9739 | NA | 0.9739 | NA | NA | NA | NA |
| MA+P+SC:MA+SA+P+SC | 0 | -0.4743 | NA | -0.4743 | NA | NA | NA | NA |
| MA+P+SC:MA+SA+SC | 0 | -0.6761 | NA | -0.6761 | NA | NA | NA | NA |
| MA+P+SC:MA+SC | 0 | 0.1603 | NA | 0.1603 | NA | NA | NA | NA |
| MA+P+SC:P+SC | 2 | 0.3946 | 0.4539 | 0.2223 | 0.2316 | -1.0017 | 1.4649 | 0.7128 |
| MA+P+SC:SA+P+SC | 0 | -1.1644 | NA | -1.1644 | NA | NA | NA | NA |
| MA+P+SC:SA+SC | 0 | -0.2296 | NA | -0.2296 | NA | NA | NA | NA |
| MA+P+SC:SC | 1 | 0.9775 | 0.8641 | 1.0957 | -0.2316 | -1.4649 | 1.0017 | 0.7128 |
| MA+SA+P+SC:MA+SA+SC | 0 | -0.2018 | NA | -0.2018 | NA | NA | NA | NA |
| MA+SA+P+SC:MA+SC | 0 | 0.6346 | NA | 0.6346 | NA | NA | NA | NA |
| MA+SA+P+SC:P+SC | 3 | 0.8689 | 0.8689 | NA | NA | NA | NA | NA |
| MA+SA+P+SC:SA+P+SC | 0 | -0.6901 | NA | -0.6901 | NA | NA | NA | NA |
| MA+SA+P+SC:SA+SC | 0 | 0.2447 | NA | 0.2447 | NA | NA | NA | NA |
| MA+SA+P+SC:SC | 0 | 1.4518 | NA | 1.4518 | NA | NA | NA | NA |
| MA+SA+SC:MA+SC | 3 | 0.8364 | 1.2804 | 0.3062 | 0.9742 | 0.1947 | 1.7537 | 0.0143 |
| MA+SA+SC:P+SC | 5 | 1.0707 | 0.7882 | 1.7053 | -0.9171 | -1.6639 | -0.1702 | 0.0161 |
| MA+SA+SC:SA+P+SC | 0 | -0.4883 | NA | -0.4883 | NA | NA | NA | NA |
| MA+SA+SC:SA+SC | 0 | 0.4465 | NA | 0.4465 | NA | NA | NA | NA |
| MA+SA+SC:SC | 2 | 1.6536 | 2.0031 | 0.9094 | 1.0937 | -0.0539 | 2.2413 | 0.0618 |
| MA+SC:P+SC | 5 | 0.2343 | 0.5214 | -0.3718 | 0.8932 | 0.1741 | 1.6122 | 0.0149 |
| MA+SC:SA+P+SC | 0 | -1.3247 | NA | -1.3247 | NA | NA | NA | NA |
| MA+SC:SA+SC | 1 | -0.3899 | -0.4895 | -0.3453 | -0.1442 | -1.2772 | 0.9888 | 0.8030 |
| MA+SC:SC | 2 | 0.8172 | 0.6120 | 1.3104 | -0.6984 | -1.8524 | 0.4556 | 0.2356 |
| P+SC:SA+P+SC | 2 | -1.5590 | -1.5590 | NA | NA | NA | NA | NA |
| P+SC:SA+SC | 4 | -0.6243 | -0.6501 | -0.2261 | -0.4240 | -2.2660 | 1.4179 | 0.6518 |
| P+SC:SC | 0 | 0.5829 | NA | 0.5829 | NA | NA | NA | NA |
| SA+P+SC:SA+SC | 0 | 0.9347 | NA | 0.9347 | NA | NA | NA | NA |
| SA+P+SC:SC | 0 | 2.1419 | NA | 2.1419 | NA | NA | NA | NA |
| SA+SC:SC | 0 | 1.2071 | NA | 1.2071 | NA | NA | NA | NA |

**Supplementary Table 8. Assessment of inconsistency using the node-splitting model in consistency-verified network meta-analysis**

| **Comparison** | **No. Studies** | **NMA** | **Direct** | **Indirect** | **Difference** | **Diff 95CI lower** | **Diff 95CI upper** | **pValue** |
| --- | --- | --- | --- | --- | --- | --- | --- | --- |
| AA+SA+SC:EA+MA+P+SC | 0 | -0.2929 | NA | -0.2929 | NA | NA | NA | NA |
| AA+SA+SC:EA+MA+SC | 0 | -0.2047 | NA | -0.2047 | NA | NA | NA | NA |
| AA+SA+SC:EA+SA+P+SC | 0 | 0.4008 | NA | 0.4008 | NA | NA | NA | NA |
| AA+SA+SC:EA+SA+SC | 0 | 0.3590 | NA | 0.3590 | NA | NA | NA | NA |
| AA+SA+SC:MA+P+SC | 0 | 0.3225 | NA | 0.3225 | NA | NA | NA | NA |
| AA+SA+SC:MA+SA+P+SC | 0 | -0.1137 | NA | -0.1137 | NA | NA | NA | NA |
| AA+SA+SC:MA+SA+SC | 0 | -0.5531 | NA | -0.5531 | NA | NA | NA | NA |
| AA+SA+SC:MA+SC | 1 | 0.4751 | 0.5042 | 0.3583 | 0.1459 | -1.8197 | 2.1115 | 0.8843 |
| AA+SA+SC:P+SC | 1 | 0.7517 | 0.7226 | 0.8683 | -0.1457 | -2.1085 | 1.8171 | 0.8843 |
| AA+SA+SC:SA+P+SC | 0 | -0.8087 | NA | -0.8087 | NA | NA | NA | NA |
| AA+SA+SC:SA+SC | 0 | 0.2523 | NA | 0.2523 | NA | NA | NA | NA |
| AA+SA+SC:SC | 0 | 1.2293 | NA | 1.2293 | NA | NA | NA | NA |
| EA+MA+P+SC:EA+MA+SC | 0 | 0.0881 | NA | 0.0881 | NA | NA | NA | NA |
| EA+MA+P+SC:EA+SA+P+SC | 0 | 0.6937 | NA | 0.6937 | NA | NA | NA | NA |
| EA+MA+P+SC:EA+SA+SC | 0 | 0.6519 | NA | 0.6519 | NA | NA | NA | NA |
| EA+MA+P+SC:MA+P+SC | 0 | 0.6154 | NA | 0.6154 | NA | NA | NA | NA |
| EA+MA+P+SC:MA+SA+P+SC | 0 | 0.1792 | NA | 0.1792 | NA | NA | NA | NA |
| EA+MA+P+SC:MA+SA+SC | 0 | -0.2602 | NA | -0.2602 | NA | NA | NA | NA |
| EA+MA+P+SC:MA+SC | 0 | 0.7679 | NA | 0.7679 | NA | NA | NA | NA |
| EA+MA+P+SC:P+SC | 1 | 1.0446 | 1.0446 | NA | NA | NA | NA | NA |
| EA+MA+P+SC:SA+P+SC | 0 | -0.5158 | NA | -0.5158 | NA | NA | NA | NA |
| EA+MA+P+SC:SA+SC | 0 | 0.5452 | NA | 0.5452 | NA | NA | NA | NA |
| EA+MA+P+SC:SC | 0 | 1.5222 | NA | 1.5222 | NA | NA | NA | NA |
| EA+MA+SC:EA+SA+P+SC | 0 | 0.6055 | NA | 0.6055 | NA | NA | NA | NA |
| EA+MA+SC:EA+SA+SC | 0 | 0.5638 | NA | 0.5638 | NA | NA | NA | NA |
| EA+MA+SC:MA+P+SC | 0 | 0.5273 | NA | 0.5273 | NA | NA | NA | NA |
| EA+MA+SC:MA+SA+P+SC | 0 | 0.0911 | NA | 0.0911 | NA | NA | NA | NA |
| EA+MA+SC:MA+SA+SC | 0 | -0.3483 | NA | -0.3483 | NA | NA | NA | NA |
| EA+MA+SC:MA+SC | 0 | 0.6798 | NA | 0.6798 | NA | NA | NA | NA |
| EA+MA+SC:P+SC | 2 | 0.9565 | 0.9565 | NA | NA | NA | NA | NA |
| EA+MA+SC:SA+P+SC | 0 | -0.6040 | NA | -0.6040 | NA | NA | NA | NA |
| EA+MA+SC:SA+SC | 0 | 0.4570 | NA | 0.4570 | NA | NA | NA | NA |
| EA+MA+SC:SC | 0 | 1.4340 | NA | 1.4340 | NA | NA | NA | NA |
| EA+SA+P+SC:EA+SA+SC | 1 | -0.0418 | 0.1109 | -0.3127 | 0.4236 | -1.0837 | 1.9308 | 0.5818 |
| EA+SA+P+SC:MA+P+SC | 0 | -0.0783 | NA | -0.0783 | NA | NA | NA | NA |
| EA+SA+P+SC:MA+SA+P+SC | 0 | -0.5145 | NA | -0.5145 | NA | NA | NA | NA |
| EA+SA+P+SC:MA+SA+SC | 0 | -0.9539 | NA | -0.9539 | NA | NA | NA | NA |
| EA+SA+P+SC:MA+SC | 0 | 0.0743 | NA | 0.0743 | NA | NA | NA | NA |
| EA+SA+P+SC:P+SC | 2 | 0.3509 | 0.2454 | 1.9137 | -1.6683 | -4.0795 | 0.7428 | 0.1751 |
| EA+SA+P+SC:SA+P+SC | 0 | -1.2095 | NA | -1.2095 | NA | NA | NA | NA |
| EA+SA+P+SC:SA+SC | 0 | -0.1485 | NA | -0.1485 | NA | NA | NA | NA |
| EA+SA+P+SC:SC | 0 | 0.8285 | NA | 0.8285 | NA | NA | NA | NA |
| EA+SA+SC:MA+P+SC | 0 | -0.0365 | NA | -0.0365 | NA | NA | NA | NA |
| EA+SA+SC:MA+SA+P+SC | 0 | -0.4727 | NA | -0.4727 | NA | NA | NA | NA |
| EA+SA+SC:MA+SA+SC | 0 | -0.9121 | NA | -0.9121 | NA | NA | NA | NA |
| EA+SA+SC:MA+SC | 0 | 0.1160 | NA | 0.1160 | NA | NA | NA | NA |
| EA+SA+SC:P+SC | 2 | 0.3927 | 0.3505 | 0.9377 | -0.5871 | -2.9560 | 1.7817 | 0.6271 |
| EA+SA+SC:SA+P+SC | 0 | -1.1677 | NA | -1.1677 | NA | NA | NA | NA |
| EA+SA+SC:SA+SC | 0 | -0.1067 | NA | -0.1067 | NA | NA | NA | NA |
| EA+SA+SC:SC | 0 | 0.8703 | NA | 0.8703 | NA | NA | NA | NA |
| MA+P+SC:MA+SA+P+SC | 0 | -0.4362 | NA | -0.4362 | NA | NA | NA | NA |
| MA+P+SC:MA+SA+SC | 0 | -0.8756 | NA | -0.8756 | NA | NA | NA | NA |
| MA+P+SC:MA+SC | 0 | 0.1525 | NA | 0.1525 | NA | NA | NA | NA |
| MA+P+SC:P+SC | 2 | 0.4292 | 0.4516 | 0.3605 | 0.0911 | -1.0989 | 1.2811 | 0.8807 |
| MA+P+SC:SA+P+SC | 0 | -1.1312 | NA | -1.1312 | NA | NA | NA | NA |
| MA+P+SC:SA+SC | 0 | -0.0702 | NA | -0.0702 | NA | NA | NA | NA |
| MA+P+SC:SC | 1 | 0.9068 | 0.8641 | 0.9552 | -0.0911 | -1.2811 | 1.0989 | 0.8807 |
| MA+SA+P+SC:MA+SA+SC | 0 | -0.4394 | NA | -0.4394 | NA | NA | NA | NA |
| MA+SA+P+SC:MA+SC | 0 | 0.5887 | NA | 0.5887 | NA | NA | NA | NA |
| MA+SA+P+SC:P+SC | 3 | 0.8654 | 0.8654 | NA | NA | NA | NA | NA |
| MA+SA+P+SC:SA+P+SC | 0 | -0.6950 | NA | -0.6950 | NA | NA | NA | NA |
| MA+SA+P+SC:SA+SC | 0 | 0.3660 | NA | 0.3660 | NA | NA | NA | NA |
| MA+SA+P+SC:SC | 0 | 1.3430 | NA | 1.3430 | NA | NA | NA | NA |
| MA+SA+SC:MA+SC | 3 | 1.0281 | 1.2813 | 0.5032 | 0.7781 | -0.0983 | 1.6545 | 0.0818 |
| MA+SA+SC:P+SC | 3 | 1.3048 | 1.0400 | 1.7264 | -0.6864 | -1.5126 | 0.1399 | 0.1035 |
| MA+SA+SC:SA+P+SC | 0 | -0.2556 | NA | -0.2556 | NA | NA | NA | NA |
| MA+SA+SC:SA+SC | 0 | 0.8054 | NA | 0.8054 | NA | NA | NA | NA |
| MA+SA+SC:SC | 2 | 1.7824 | 2.0010 | 1.2531 | 0.7479 | -0.3927 | 1.8885 | 0.1987 |
| MA+SC:P+SC | 3 | 0.2767 | 0.5170 | -0.1142 | 0.6312 | -0.1878 | 1.4503 | 0.1309 |
| MA+SC:SA+P+SC | 0 | -1.2838 | NA | -1.2838 | NA | NA | NA | NA |
| MA+SC:SA+SC | 0 | -0.2228 | NA | -0.2228 | NA | NA | NA | NA |
| MA+SC:SC | 2 | 0.7543 | 0.6123 | 1.1460 | -0.5338 | -1.6823 | 0.6148 | 0.3624 |
| P+SC:SA+P+SC | 2 | -1.5604 | -1.5604 | NA | NA | NA | NA | NA |
| P+SC:SA+SC | 3 | -0.4994 | -0.4994 | NA | NA | NA | NA | NA |
| P+SC:SC | 0 | 0.4776 | NA | 0.4776 | NA | NA | NA | NA |
| SA+P+SC:SA+SC | 0 | 1.0610 | NA | 1.0610 | NA | NA | NA | NA |
| SA+P+SC:SC | 0 | 2.0380 | NA | 2.0380 | NA | NA | NA | NA |
| SA+SC:SC | 0 | 0.9770 | NA | 0.9770 | NA | NA | NA | NA |

**Supplementary Table 9. League table of the subgroup network meta-analysis on cognitive status in patients with vascular dementia**

Pairwise (upper-right portion) and network (lower-left portion) meta-analysis results are presented as estimate effect sizes for the outcome of changes of cognitive status outcomes in patients with VCI. Outcomes are presented as standardized mean difference (SMD) (95% confidence intervals). For the pairwise meta-analyses, SMD of more than 0 indicate that the treatment specified in the row got more beneficial effect than that specified in the column. For the network meta-analysis (NMA), SMD of more than 0 indicate that the treatment specified in the column got more beneficial effect than that specified in the row. Grey grids: treatment. Blue grids: treatment in column is significantly more effective than treatment in row. Green grids: treatment in row is significantly more effective than treatment in column. Abbreviations: AA, auricular acupuncture; CR, cognitive rehabilitation; EA, electroacupuncture; MA, manual acupuncture; P, pharmacotherapy; SA, scalp acupuncture; SC, standard care.

| **SA+P+SC** | . | . | . | . | . | . | . | . | . | . | 1.88 (1.11, 2.66) | . |
| --- | --- | --- | --- | --- | --- | --- | --- | --- | --- | --- | --- | --- |
| 0.70 (-0.17, 1.57) | **MA+SA+SC** | . | . | . | . | . | . | . | . | 1.28 (0.84, 1.73) | 1.05 (0.58, 1.51) | 2.00 (1.44, 2.55) |
| 0.81 (-0.31, 1.93) | 0.11 (-0.78, 1.00) | **MA+SA+P+SC** | . | . | . | . | . | . | . | . | 1.08 (0.27, 1.88) | . |
| 0.84 (-0.28, 1.96) | 0.14 (-0.75, 1.03) | 0.03 (-1.10, 1.17) | **EA+MA+P+SC** | . | . | . | . | . | . | . | 1.04 (0.24, 1.85) | . |
| 0.93 (-0.03, 1.89) | 0.23 (-0.45, 0.92) | 0.12 (-0.86, 1.11) | 0.09 (-0.89, 1.07) | **EA+MA+SC** | . | . | . | . | . | . | 0.96 (0.39, 1.52) | . |
| 1.25 (0.19, 2.30) | 0.55 (-0.20, 1.30) | 0.44 (-0.63, 1.52) | 0.41 (-0.67, 1.48) | 0.32 (-0.59, 1.23) | **AA+SA+SC** | . | . | . | . | 0.50 (-0.28, 1.29) | 0.72 (-0.07, 1.51) | . |
| 1.38 (0.49, 2.28) | 0.69 (0.10, 1.27) | 0.58 (-0.34, 1.49) | 0.54 (-0.37, 1.46) | 0.45 (-0.26, 1.17) | 0.14 (-0.70, 0.97) | **SA+SC** | . | . | . | . | 0.50 (0.06, 0.94) | . |
| 1.44 (0.50, 2.38) | 0.74 (0.09, 1.39) | 0.63 (-0.33, 1.59) | 0.60 (-0.36, 1.56) | 0.51 (-0.26, 1.28) | 0.19 (-0.69, 1.08) | 0.06 (-0.63, 0.74) | **MA+P+SC** | . | . | . | 0.45 (-0.08, 0.97) | . |
| 1.49 (0.53, 2.44) | 0.79 (0.12, 1.46) | 0.68 (-0.29, 1.66) | 0.65 (-0.33, 1.62) | 0.56 (-0.23, 1.35) | 0.24 (-0.66, 1.14) | 0.10 (-0.60, 0.81) | 0.05 (-0.71, 0.81) | **EA+SA+SC** | -0.11 (-0.93, 0.71) | . | 0.35 (-0.22, 0.93) | . |
| 1.53 (0.59, 2.47) | 0.83 (0.18, 1.48) | 0.72 (-0.24, 1.68) | 0.69 (-0.27, 1.65) | 0.60 (-0.17, 1.37) | 0.28 (-0.60, 1.17) | 0.15 (-0.54, 0.83) | 0.09 (-0.65, 0.83) | 0.04 (-0.61, 0.69) | **EA+SA+P+SC** | . | 0.25 (-0.29, 0.79) | . |
| 1.84 (0.95, 2.72) | 1.14 (0.76, 1.52) | 1.03 (0.12, 1.94) | 1.00 (0.09, 1.90) | 0.91 (0.20, 1.61) | 0.59 (-0.12, 1.30) | 0.45 (-0.15, 1.06) | 0.40 (-0.27, 1.07) | 0.35 (-0.34, 1.04) | 0.31 (-0.36, 0.98) | **MA+SC** | 0.25 (-0.30, 0.81) | 0.61 (0.09, 1.14) |
| 1.88 (1.11, 2.66) | 1.19 (0.80, 1.57) | 1.08 (0.27, 1.88) | 1.04 (0.24, 1.85) | 0.96 (0.39, 1.52) | 0.64 (-0.08, 1.35) | 0.50 (0.06, 0.94) | 0.45 (-0.08, 0.97) | 0.40 (-0.15, 0.95) | 0.35 (-0.17, 0.88) | 0.05 (-0.37, 0.47) | **P+SC** | . |
| 2.55 (1.58, 3.53) | 1.85 (1.34, 2.37) | 1.75 (0.75, 2.74) | 1.71 (0.72, 2.71) | 1.62 (0.81, 2.44) | 1.30 (0.46, 2.15) | 1.17 (0.43, 1.90) | 1.11 (0.33, 1.90) | 1.06 (0.26, 1.87) | 1.02 (0.23, 1.81) | 0.71 (0.22, 1.21) | 0.67 (0.08, 1.26) | **SC** |

**Supplementary Table 10. Assessment of inconsistency using the node-splitting model in subgroup analysis**

| **Comparison** | **No. Studies** | **NMA** | **Direct** | **Indirect** | **Difference** | **Diff 95CI lower** | **Diff 95CI upper** | **pValue** |
| --- | --- | --- | --- | --- | --- | --- | --- | --- |
| AA+SA+SC:EA+MA+P+SC | 0 | -0.4076 | NA | -0.4076 | NA | NA | NA | NA |
| AA+SA+SC:EA+MA+SC | 0 | -0.3183 | NA | -0.3183 | NA | NA | NA | NA |
| AA+SA+SC:EA+SA+P+SC | 0 | 0.2820 | NA | 0.2820 | NA | NA | NA | NA |
| AA+SA+SC:EA+SA+SC | 0 | 0.2401 | NA | 0.2401 | NA | NA | NA | NA |
| AA+SA+SC:MA+P+SC | 0 | 0.1913 | NA | 0.1913 | NA | NA | NA | NA |
| AA+SA+SC:MA+SA+P+SC | 0 | -0.4413 | NA | -0.4413 | NA | NA | NA | NA |
| AA+SA+SC:MA+SA+SC | 0 | -0.5498 | NA | -0.5498 | NA | NA | NA | NA |
| AA+SA+SC:MA+SC | 1 | 0.5895 | 0.5042 | 0.9786 | -0.4744 | -2.3321 | 1.3833 | 0.6167 |
| AA+SA+SC:P+SC | 1 | 0.6370 | 0.7226 | 0.2490 | 0.4736 | -1.3808 | 2.3280 | 0.6167 |
| AA+SA+SC:SA+P+SC | 0 | -1.2469 | NA | -1.2469 | NA | NA | NA | NA |
| AA+SA+SC:SA+SC | 0 | 0.1358 | NA | 0.1358 | NA | NA | NA | NA |
| AA+SA+SC:SC | 0 | 1.3042 | NA | 1.3042 | NA | NA | NA | NA |
| EA+MA+P+SC:EA+MA+SC | 0 | 0.0893 | NA | 0.0893 | NA | NA | NA | NA |
| EA+MA+P+SC:EA+SA+P+SC | 0 | 0.6897 | NA | 0.6897 | NA | NA | NA | NA |
| EA+MA+P+SC:EA+SA+SC | 0 | 0.6477 | NA | 0.6477 | NA | NA | NA | NA |
| EA+MA+P+SC:MA+P+SC | 0 | 0.5989 | NA | 0.5989 | NA | NA | NA | NA |
| EA+MA+P+SC:MA+SA+P+SC | 0 | -0.0337 | NA | -0.0337 | NA | NA | NA | NA |
| EA+MA+P+SC:MA+SA+SC | 0 | -0.1422 | NA | -0.1422 | NA | NA | NA | NA |
| EA+MA+P+SC:MA+SC | 0 | 0.9971 | NA | 0.9971 | NA | NA | NA | NA |
| EA+MA+P+SC:P+SC | 1 | 1.0446 | 1.0446 | NA | NA | NA | NA | NA |
| EA+MA+P+SC:SA+P+SC | 0 | -0.8393 | NA | -0.8393 | NA | NA | NA | NA |
| EA+MA+P+SC:SA+SC | 0 | 0.5434 | NA | 0.5434 | NA | NA | NA | NA |
| EA+MA+P+SC:SC | 0 | 1.7118 | NA | 1.7118 | NA | NA | NA | NA |
| EA+MA+SC:EA+SA+P+SC | 0 | 0.6003 | NA | 0.6003 | NA | NA | NA | NA |
| EA+MA+SC:EA+SA+SC | 0 | 0.5584 | NA | 0.5584 | NA | NA | NA | NA |
| EA+MA+SC:MA+P+SC | 0 | 0.5096 | NA | 0.5096 | NA | NA | NA | NA |
| EA+MA+SC:MA+SA+P+SC | 0 | -0.1230 | NA | -0.1230 | NA | NA | NA | NA |
| EA+MA+SC:MA+SA+SC | 0 | -0.2315 | NA | -0.2315 | NA | NA | NA | NA |
| EA+MA+SC:MA+SC | 0 | 0.9078 | NA | 0.9078 | NA | NA | NA | NA |
| EA+MA+SC:P+SC | 2 | 0.9553 | 0.9553 | NA | NA | NA | NA | NA |
| EA+MA+SC:SA+P+SC | 0 | -0.9286 | NA | -0.9286 | NA | NA | NA | NA |
| EA+MA+SC:SA+SC | 0 | 0.4541 | NA | 0.4541 | NA | NA | NA | NA |
| EA+MA+SC:SC | 0 | 1.6225 | NA | 1.6225 | NA | NA | NA | NA |
| EA+SA+P+SC:EA+SA+SC | 1 | -0.0419 | 0.1109 | -0.3053 | 0.4162 | -0.9335 | 1.7659 | 0.5456 |
| EA+SA+P+SC:MA+P+SC | 0 | -0.0908 | NA | -0.0908 | NA | NA | NA | NA |
| EA+SA+P+SC:MA+SA+P+SC | 0 | -0.7233 | NA | -0.7233 | NA | NA | NA | NA |
| EA+SA+P+SC:MA+SA+SC | 0 | -0.8318 | NA | -0.8318 | NA | NA | NA | NA |
| EA+SA+P+SC:MA+SC | 0 | 0.3074 | NA | 0.3074 | NA | NA | NA | NA |
| EA+SA+P+SC:P+SC | 2 | 0.3549 | 0.2519 | 1.9069 | -1.6550 | -3.8204 | 0.5105 | 0.1342 |
| EA+SA+P+SC:SA+P+SC | 0 | -1.5290 | NA | -1.5290 | NA | NA | NA | NA |
| EA+SA+P+SC:SA+SC | 0 | -0.1463 | NA | -0.1463 | NA | NA | NA | NA |
| EA+SA+P+SC:SC | 0 | 1.0221 | NA | 1.0221 | NA | NA | NA | NA |
| EA+SA+SC:MA+P+SC | 0 | -0.0488 | NA | -0.0488 | NA | NA | NA | NA |
| EA+SA+SC:MA+SA+P+SC | 0 | -0.6814 | NA | -0.6814 | NA | NA | NA | NA |
| EA+SA+SC:MA+SA+SC | 0 | -0.7899 | NA | -0.7899 | NA | NA | NA | NA |
| EA+SA+SC:MA+SC | 0 | 0.3494 | NA | 0.3494 | NA | NA | NA | NA |
| EA+SA+SC:P+SC | 2 | 0.3969 | 0.3540 | 0.9398 | -0.5858 | -2.7033 | 1.5318 | 0.5877 |
| EA+SA+SC:SA+P+SC | 0 | -1.4870 | NA | -1.4870 | NA | NA | NA | NA |
| EA+SA+SC:SA+SC | 0 | -0.1043 | NA | -0.1043 | NA | NA | NA | NA |
| EA+SA+SC:SC | 0 | 1.0641 | NA | 1.0641 | NA | NA | NA | NA |
| MA+P+SC:MA+SA+P+SC | 0 | -0.6326 | NA | -0.6326 | NA | NA | NA | NA |
| MA+P+SC:MA+SA+SC | 0 | -0.7411 | NA | -0.7411 | NA | NA | NA | NA |
| MA+P+SC:MA+SC | 0 | 0.3982 | NA | 0.3982 | NA | NA | NA | NA |
| MA+P+SC:P+SC | 2 | 0.4457 | 0.4457 | NA | NA | NA | NA | NA |
| MA+P+SC:SA+P+SC | 0 | -1.4382 | NA | -1.4382 | NA | NA | NA | NA |
| MA+P+SC:SA+SC | 0 | -0.0555 | NA | -0.0555 | NA | NA | NA | NA |
| MA+P+SC:SC | 0 | 1.1129 | NA | 1.1129 | NA | NA | NA | NA |
| MA+SA+P+SC:MA+SA+SC | 0 | -0.1085 | NA | -0.1085 | NA | NA | NA | NA |
| MA+SA+P+SC:MA+SC | 0 | 1.0307 | NA | 1.0307 | NA | NA | NA | NA |
| MA+SA+P+SC:P+SC | 1 | 1.0782 | 1.0782 | NA | NA | NA | NA | NA |
| MA+SA+P+SC:SA+P+SC | 0 | -0.8057 | NA | -0.8057 | NA | NA | NA | NA |
| MA+SA+P+SC:SA+SC | 0 | 0.5770 | NA | 0.5770 | NA | NA | NA | NA |
| MA+SA+P+SC:SC | 0 | 1.7454 | NA | 1.7454 | NA | NA | NA | NA |
| MA+SA+SC:MA+SC | 3 | 1.1393 | 1.2836 | 0.7629 | 0.5207 | -0.3283 | 1.3696 | 0.2293 |
| MA+SA+SC:P+SC | 3 | 1.1868 | 1.0457 | 1.5244 | -0.4787 | -1.3281 | 0.3707 | 0.2693 |
| MA+SA+SC:SA+P+SC | 0 | -0.6971 | NA | -0.6971 | NA | NA | NA | NA |
| MA+SA+SC:SA+SC | 0 | 0.6856 | NA | 0.6856 | NA | NA | NA | NA |
| MA+SA+SC:SC | 2 | 1.8540 | 1.9959 | 1.0062 | 0.9897 | -0.4692 | 2.4486 | 0.1837 |
| MA+SC:P+SC | 2 | 0.0475 | 0.2527 | -0.2260 | 0.4787 | -0.3707 | 1.3281 | 0.2693 |
| MA+SC:SA+P+SC | 0 | -1.8364 | NA | -1.8364 | NA | NA | NA | NA |
| MA+SC:SA+SC | 0 | -0.4537 | NA | -0.4537 | NA | NA | NA | NA |
| MA+SC:SC | 2 | 0.7147 | 0.6130 | 1.5724 | -0.9594 | -2.5727 | 0.6539 | 0.2438 |
| P+SC:SA+P+SC | 1 | -1.8839 | -1.8839 | NA | NA | NA | NA | NA |
| P+SC:SA+SC | 3 | -0.5012 | -0.5012 | NA | NA | NA | NA | NA |
| P+SC:SC | 0 | 0.6672 | NA | 0.6672 | NA | NA | NA | NA |
| SA+P+SC:SA+SC | 0 | 1.3827 | NA | 1.3827 | NA | NA | NA | NA |
| SA+P+SC:SC | 0 | 2.5511 | NA | 2.5511 | NA | NA | NA | NA |
| SA+SC:SC | 0 | 1.1684 | NA | 1.1684 | NA | NA | NA | NA |

**Supplementary Table 11. Risk of bias results assessed by RoB 2**

| **Author, Year** | **Domain 1** | **Domain 2** | **Domain 3** | **Domain 4** | **Domain 5** | **Overall risk of bias** |
| --- | --- | --- | --- | --- | --- | --- |
| Mo 2000 | Some concerns | Low risk | Low risk | Low risk | Low risk | Some concerns |
| Mo FZ 2000 | Some concerns | Low risk | Low risk | Low risk | Low risk | Some concerns |
| Niu 2007 | Low risk | Low risk | Low risk | Low risk | Low risk | Low risk |
| Chen 2009 | Low risk | Low risk | Low risk | Low risk | Low risk | Low risk |
| Meng 2009 | Some concerns | Low risk | Low risk | Low risk | Low risk | Some concerns |
| Zhao 2009 | Low risk | Low risk | Low risk | Low risk | Low risk | Low risk |
| Yin 2011 | Some concerns | Low risk | Low risk | Low risk | Low risk | Some concerns |
| Li P 2012 | Some concerns | Low risk | Low risk | Low risk | Low risk | Some concerns |
| Li S 2012 | Some concerns | Some concerns | Low risk | Low risk | Low risk | Some concerns |
| Li W 2012 | Low risk | Low risk | Low risk | Low risk | Low risk | Low risk |
| Teng 2012 | Some concerns | Low risk | Low risk | Low risk | Low risk | Some concerns |
| Lin 2012 | Low risk | Low risk | Low risk | Low risk | Low risk | Low risk |
| Zhao 2013 | Some concerns | Low risk | Low risk | Low risk | Low risk | Some concerns |
| Cao 2014 | Low risk | Low risk | Low risk | Low risk | Low risk | Low risk |
| Li 2014 | Some concerns | Low risk | Low risk | Low risk | Low risk | Some concerns |
| Li S 2014 | Low risk | Low risk | Low risk | Low risk | Low risk | Low risk |
| Cui 2015 | Some concerns | Low risk | Low risk | Low risk | Low risk | Some concerns |
| Luo 2015 | Low risk | Low risk | Low risk | Low risk | Low risk | Low risk |
| Zhang 2015 | Some concerns | Low risk | Low risk | Low risk | Low risk | Some concerns |
| Yang 2016 | Low risk | Low risk | Low risk | Low risk | Low risk | Low risk |
| Tan 2017 | Some concerns | Low risk | Low risk | Low risk | Low risk | Some concerns |
| Wang 2017 | Low risk | Low risk | Low risk | Low risk | Low risk | Low risk |
| Wang F 2017 | Low risk | Low risk | Low risk | Low risk | Low risk | Low risk |
| Cheng 2018 | Low risk | Low risk | Low risk | Low risk | Low risk | Low risk |
| Hu 2019 | Low risk | Low risk | Low risk | Low risk | Low risk | Low risk |
| Jiang 2019 | Low risk | Low risk | Low risk | Low risk | Low risk | Low risk |
| Li 2019 | Low risk | Low risk | Low risk | Low risk | Low risk | Low risk |
| Zhang 2019 | Some concerns | Low risk | Low risk | Low risk | Low risk | Some concerns |
| Chen 2020 | Some concerns | Low risk | Low risk | Low risk | Low risk | Some concerns |
| Meng 2020 | Low risk | Low risk | Some concerns | Low risk | Low risk | Some concerns |
| Qu 2020 | Low risk | Low risk | Low risk | Low risk | Low risk | Low risk |
| Gao 2021 | Low risk | Low risk | Low risk | Low risk | Low risk | Low risk |
| Zhou 2022 | Some concerns | Low risk | Low risk | Low risk | Low risk | Some concerns |
| Shen 2022 | Low risk | Low risk | Low risk | Low risk | Low risk | Low risk |
| Wang 2022 | Some concerns | Low risk | Low risk | Low risk | Low risk | Some concerns |
| Chen 2022 | Low risk | Low risk | Low risk | Low risk | Low risk | Low risk |
| Qiao 2023 | Some concerns | Low risk | Low risk | Low risk | Low risk | Some concerns |
| Zhang 2024 | Some concerns | Low risk | Low risk | Low risk | Low risk | Some concerns |
| Liu 2025 | Low risk | Low risk | Low risk | Low risk | Low risk | Low risk |
| Sun 2025 | Low risk | Low risk | Low risk | Low risk | Low risk | Low risk |

Domain 1: Bias arising from the randomization process

Domain 2: Bias due to deviations from the intended interventions

Domain 3: Bias due to missing outcome data

Domain 4: Bias in measurement of the outcome

Domain 5: Bias in selection of the reported result

**Supplementary Table 12. Specific reasons for "some concerns" in included studies**

| **Author, Year** | **Domain 1** | **Domain 2** | **Domain 3** |
| --- | --- | --- | --- |
| Mo 2000 | Unclear randomization of the allocation sequence | - | - |
| Mo FZ 2000 | Unclear randomization of the allocation sequence | - | - |
| Meng 2009 | Unclear allocation sequence concealment until participants enrolled | - | - |
| Yin 2011 | Unclear randomization of the allocation sequence | - | - |
| Li P 2012 | Unclear randomization of the allocation sequence | - | - |
| Li S 2012 | Unclear allocation sequence concealment until participants enrolled | Participants aware of their assigned intervention during the trial | - |
| Teng 2012 | Unclear randomization of the allocation sequence | - | - |
| Zhao 2013 | Unclear randomization of the allocation sequence | - | - |
| Li 2014 | Unclear randomization of the allocation sequence | - | - |
| Cui 2015 | Unclear allocation sequence concealment until participants enrolled | - | - |
| Zhang 2015 | Unclear allocation sequence concealment until participants enrolled | - | - |
| Tan 2017 | Unclear randomization of the allocation sequence | - | - |
| Zhang 2019 | Unclear randomization of the allocation sequence | - | - |
| Chen 2020 | Unclear randomization of the allocation sequence | - | - |
| Meng 2020 | - | - | Unclear description of intervention or regimen that might affect the outcome |
| Zhou 2022 | Unclear randomization of the allocation sequence | - | - |
| Wang 2022 | Unclear randomization of the allocation sequence | - | - |
| Qiao 2023 | Unclear randomization of the allocation sequence | - | - |
| Zhang 2024 | Unclear randomization of the allocation sequence | - | - |

**Supplementary Table 13. CINeMA assessment report**

| **Comparison** | **Number of studies** | **Within-study bias** | **Reporting bias** | **Indirectness** | **Imprecision** | **Heterogeneity** | **Incoherence** | **Confidence rating** |
| --- | --- | --- | --- | --- | --- | --- | --- | --- |
| **AA+SA+SC:MA+SC** | 1 | No concerns | Low risk | No concerns | Some concerns | Some concerns | No concerns | Low |
| **AA+SA+SC:P+SC** | 1 | No concerns | Low risk | No concerns | Some concerns | No concerns | No concerns | Moderate |
| **EA+MA+P+SC:P+SC** | 1 | No concerns | Low risk | No concerns | No concerns | Some concerns | No concerns | Moderate |
| **EA+MA+SC:P+SC** | 2 | Some concerns | Low risk | No concerns | No concerns | Some concerns | No concerns | Low |
| **EA+SA+P+SC:EA+SA+SC** | 1 | No concerns | Low risk | No concerns | Major concerns | No concerns | No concerns | Low |
| **EA+SA+P+SC:P+SC** | 2 | No concerns | Low risk | No concerns | Some concerns | Some concerns | No concerns | Low |
| **EA+SA+SC:P+SC** | 2 | No concerns | Low risk | No concerns | Some concerns | Some concerns | No concerns | Low |
| **MA+P+SC:P+SC** | 2 | No concerns | Low risk | No concerns | Some concerns | No concerns | No concerns | Moderate |
| **MA+P+SC:SC** | 1 | Some concerns | Low risk | No concerns | No concerns | Some concerns | No concerns | Low |
| **MA+SA+P+SC:P+SC** | 3 | Some concerns | Low risk | No concerns | No concerns | Some concerns | No concerns | Low |
| **MA+SA+SC:MA+SC** | 3 | No concerns | Low risk | No concerns | No concerns | No concerns | Some concerns | Moderate |
| **MA+SA+SC:P+SC** | 3 | Some concerns | Low risk | No concerns | No concerns | No concerns | No concerns | Moderate |
| **MA+SA+SC:SC** | 2 | No concerns | Low risk | No concerns | No concerns | No concerns | No concerns | High |
| **MA+SC:P+SC** | 3 | No concerns | Low risk | No concerns | Some concerns | Some concerns | No concerns | Low |
| **MA+SC:SC** | 2 | No concerns | Low risk | No concerns | No concerns | Some concerns | No concerns | Moderate |
| **P+SC:SA+P+SC** | 2 | No concerns | Low risk | No concerns | No concerns | No concerns | No concerns | High |
| **P+SC:SA+SC** | 3 | No concerns | Low risk | No concerns | No concerns | Some concerns | No concerns | Moderate |
| **AA+SA+SC:EA+MA+P+SC** | 0 | No concerns | Low risk | No concerns | Major concerns | No concerns | No concerns | Low |
| **AA+SA+SC:EA+MA+SC** | 0 | No concerns | Low risk | No concerns | Major concerns | No concerns | No concerns | Low |
| **AA+SA+SC:EA+SA+P+SC** | 0 | No concerns | Low risk | No concerns | Major concerns | No concerns | No concerns | Low |
| **AA+SA+SC:EA+SA+SC** | 0 | No concerns | Low risk | No concerns | Major concerns | No concerns | No concerns | Low |
| **AA+SA+SC:MA+P+SC** | 0 | No concerns | Low risk | No concerns | Major concerns | No concerns | No concerns | Low |
| **AA+SA+SC:MA+SA+P+SC** | 0 | No concerns | Low risk | No concerns | Major concerns | No concerns | No concerns | Low |
| **AA+SA+SC:MA+SA+SC** | 0 | No concerns | Low risk | No concerns | Some concerns | Some concerns | No concerns | Low |
| **AA+SA+SC:SA+P+SC** | 0 | No concerns | Low risk | No concerns | Some concerns | No concerns | No concerns | Moderate |
| **AA+SA+SC:SA+SC** | 0 | No concerns | Low risk | No concerns | Major concerns | No concerns | No concerns | Low |
| **AA+SA+SC:SC** | 0 | No concerns | Low risk | No concerns | No concerns | No concerns | No concerns | High |
| **EA+MA+P+SC:EA+MA+SC** | 0 | No concerns | Low risk | No concerns | Major concerns | No concerns | No concerns | Low |
| **EA+MA+P+SC:EA+SA+P+SC** | 0 | No concerns | Low risk | No concerns | Some concerns | Some concerns | No concerns | Low |
| **EA+MA+P+SC:EA+SA+SC** | 0 | No concerns | Low risk | No concerns | Some concerns | Some concerns | No concerns | Low |
| **EA+MA+P+SC:MA+P+SC** | 0 | No concerns | Low risk | No concerns | Some concerns | Some concerns | No concerns | Low |
| **EA+MA+P+SC:MA+SA+P+SC** | 0 | No concerns | Low risk | No concerns | Major concerns | No concerns | No concerns | Low |
| **EA+MA+P+SC:MA+SA+SC** | 0 | No concerns | Low risk | No concerns | Major concerns | No concerns | No concerns | Low |
| **EA+MA+P+SC:MA+SC** | 0 | No concerns | Low risk | No concerns | Some concerns | Some concerns | No concerns | Low |
| **EA+MA+P+SC:SA+P+SC** | 0 | No concerns | Low risk | No concerns | Major concerns | No concerns | No concerns | Low |
| **EA+MA+P+SC:SA+SC** | 0 | No concerns | Low risk | No concerns | Some concerns | Some concerns | No concerns | Low |
| **EA+MA+P+SC:SC** | 0 | No concerns | Low risk | No concerns | No concerns | No concerns | No concerns | High |
| **EA+MA+SC:EA+SA+P+SC** | 0 | Some concerns | Low risk | No concerns | Some concerns | Some concerns | No concerns | Very low |
| **EA+MA+SC:EA+SA+SC** | 0 | Some concerns | Low risk | No concerns | Some concerns | Some concerns | No concerns | Very low |
| **EA+MA+SC:MA+P+SC** | 0 | Some concerns | Low risk | No concerns | Some concerns | Some concerns | No concerns | Very low |
| **EA+MA+SC:MA+SA+P+SC** | 0 | Some concerns | Low risk | No concerns | Major concerns | No concerns | No concerns | Low |
| **EA+MA+SC:MA+SA+SC** | 0 | Some concerns | Low risk | No concerns | Some concerns | Some concerns | No concerns | Very low |
| **EA+MA+SC:MA+SC** | 0 | Some concerns | Low risk | No concerns | Some concerns | No concerns | No concerns | Low |
| **EA+MA+SC:SA+P+SC** | 0 | Some concerns | Low risk | No concerns | Some concerns | Some concerns | No concerns | Very low |
| **EA+MA+SC:SA+SC** | 0 | Some concerns | Low risk | No concerns | Some concerns | Some concerns | No concerns | Very low |
| **EA+MA+SC:SC** | 0 | Some concerns | Low risk | No concerns | No concerns | No concerns | No concerns | Moderate |
| **EA+SA+P+SC:MA+P+SC** | 0 | No concerns | Low risk | No concerns | Major concerns | No concerns | No concerns | Low |
| **EA+SA+P+SC:MA+SA+P+SC** | 0 | No concerns | Low risk | No concerns | Some concerns | Some concerns | No concerns | Low |
| **EA+SA+P+SC:MA+SA+SC** | 0 | No concerns | Low risk | No concerns | No concerns | Some concerns | No concerns | Moderate |
| **EA+SA+P+SC:MA+SC** | 0 | No concerns | Low risk | No concerns | Major concerns | No concerns | No concerns | Low |
| **EA+SA+P+SC:SA+P+SC** | 0 | No concerns | Low risk | No concerns | No concerns | No concerns | No concerns | High |
| **EA+SA+P+SC:SA+SC** | 0 | No concerns | Low risk | No concerns | Major concerns | No concerns | No concerns | Low |
| **EA+SA+P+SC:SC** | 0 | No concerns | Low risk | No concerns | No concerns | Some concerns | No concerns | Moderate |
| **EA+SA+SC:MA+P+SC** | 0 | No concerns | Low risk | No concerns | Major concerns | No concerns | No concerns | Low |
| **EA+SA+SC:MA+SA+P+SC** | 0 | Some concerns | Low risk | No concerns | Some concerns | Some concerns | No concerns | Very low |
| **EA+SA+SC:MA+SA+SC** | 0 | Some concerns | Low risk | No concerns | No concerns | Some concerns | No concerns | Moderate |
| **EA+SA+SC:MA+SC** | 0 | No concerns | Low risk | No concerns | Major concerns | No concerns | No concerns | Low |
| **EA+SA+SC:SA+P+SC** | 0 | No concerns | Low risk | No concerns | No concerns | Some concerns | No concerns | Moderate |
| **EA+SA+SC:SA+SC** | 0 | No concerns | Low risk | No concerns | Major concerns | No concerns | No concerns | Low |
| **EA+SA+SC:SC** | 0 | No concerns | Low risk | No concerns | No concerns | Some concerns | No concerns | Moderate |
| **MA+P+SC:MA+SA+P+SC** | 0 | Some concerns | Low risk | No concerns | Some concerns | Some concerns | No concerns | Very low |
| **MA+P+SC:MA+SA+SC** | 0 | Some concerns | Low risk | No concerns | No concerns | Some concerns | No concerns | Low |
| **MA+P+SC:MA+SC** | 0 | No concerns | Low risk | No concerns | Some concerns | Some concerns | No concerns | Low |
| **MA+P+SC:SA+P+SC** | 0 | No concerns | Low risk | No concerns | No concerns | Some concerns | No concerns | Moderate |
| **MA+P+SC:SA+SC** | 0 | No concerns | Low risk | No concerns | Major concerns | No concerns | No concerns | Low |
| **MA+SA+P+SC:MA+SA+SC** | 0 | Some concerns | Low risk | No concerns | Some concerns | Some concerns | No concerns | Very low |
| **MA+SA+P+SC:MA+SC** | 0 | No concerns | Low risk | No concerns | Some concerns | No concerns | No concerns | Moderate |
| **MA+SA+P+SC:SA+P+SC** | 0 | Some concerns | Low risk | No concerns | Some concerns | No concerns | No concerns | Low |
| **MA+SA+P+SC:SA+SC** | 0 | Some concerns | Low risk | No concerns | Some concerns | Some concerns | No concerns | Very low |
| **MA+SA+P+SC:SC** | 0 | No concerns | Low risk | No concerns | No concerns | No concerns | No concerns | High |
| **MA+SA+SC:SA+P+SC** | 0 | Some concerns | Low risk | No concerns | Some concerns | Some concerns | No concerns | Very low |
| **MA+SA+SC:SA+SC** | 0 | No concerns | Low risk | No concerns | No concerns | Some concerns | No concerns | Moderate |
| **MA+SC:SA+P+SC** | 0 | No concerns | Low risk | No concerns | No concerns | No concerns | No concerns | High |
| **MA+SC:SA+SC** | 0 | No concerns | Low risk | No concerns | Some concerns | Some concerns | No concerns | Low |
| **P+SC:SC** | 0 | No concerns | Low risk | No concerns | Some concerns | No concerns | No concerns | Moderate |
| **SA+P+SC:SA+SC** | 0 | No concerns | Low risk | No concerns | No concerns | Some concerns | No concerns | Moderate |
| **SA+P+SC:SC** | 0 | No concerns | Low risk | No concerns | No concerns | No concerns | No concerns | High |
| **SA+SC:SC** | 0 | No concerns | Low risk | No concerns | No concerns | Some concerns | No concerns | Moderate |

**Supplementary Figure 1. Initial network meta-analysis results** **of the cognitive status**


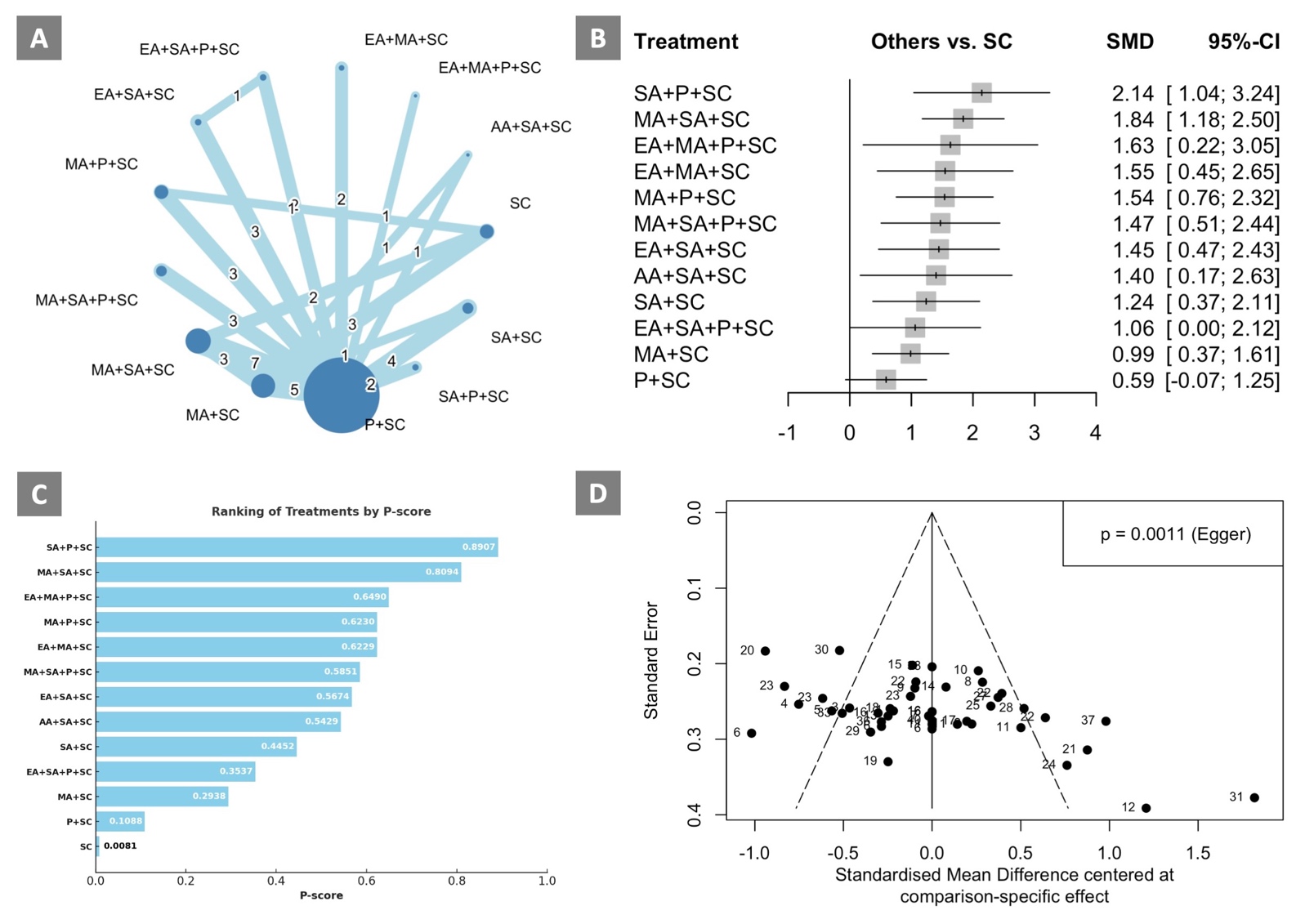


**Supplementary Figure 2. Publication bias-adjusted network meta-analysis results of the cognitive status**

**
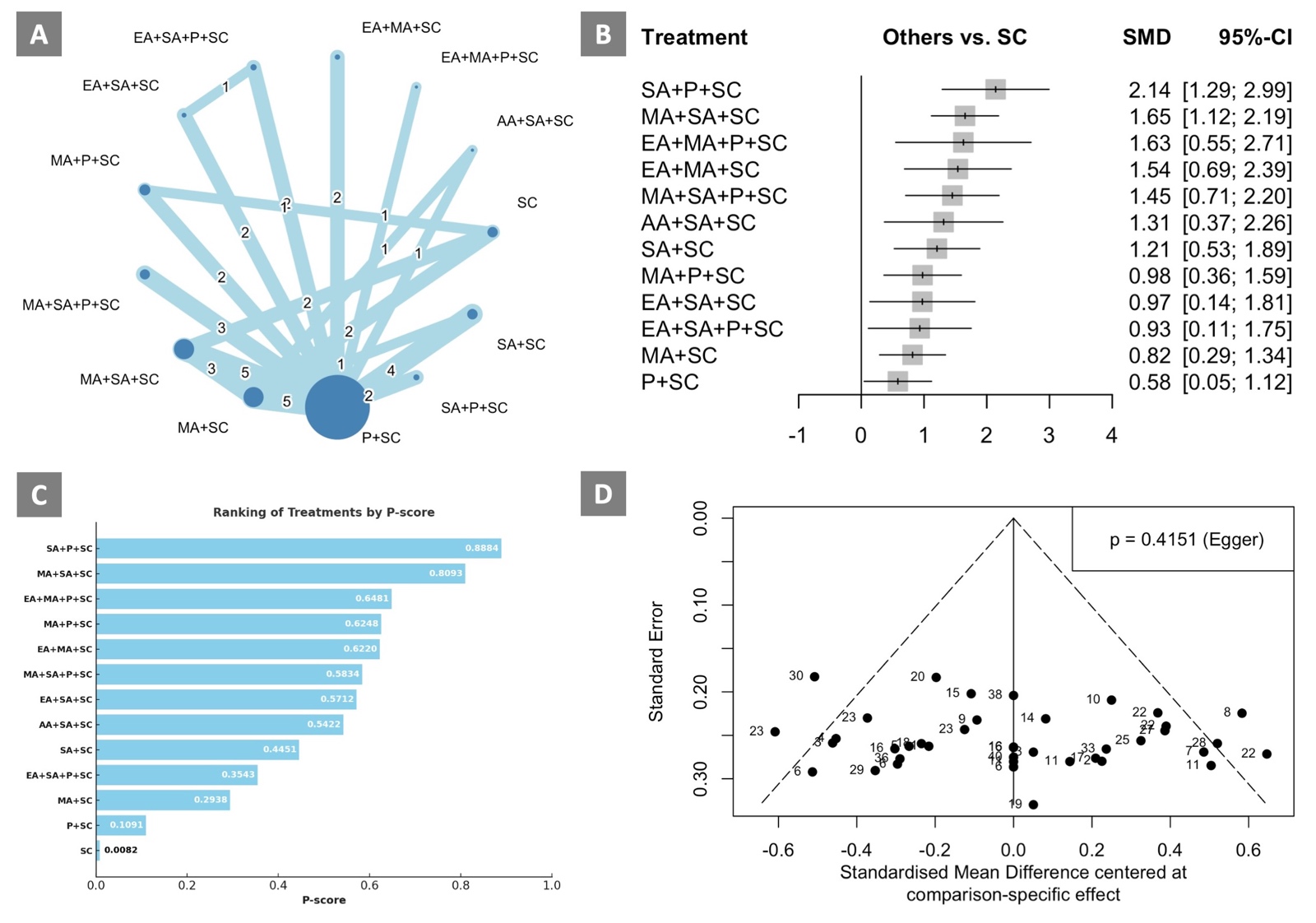
**

**Supplementary Figure 3. Subgroup network meta-analysis results of cognitive status in VD patients
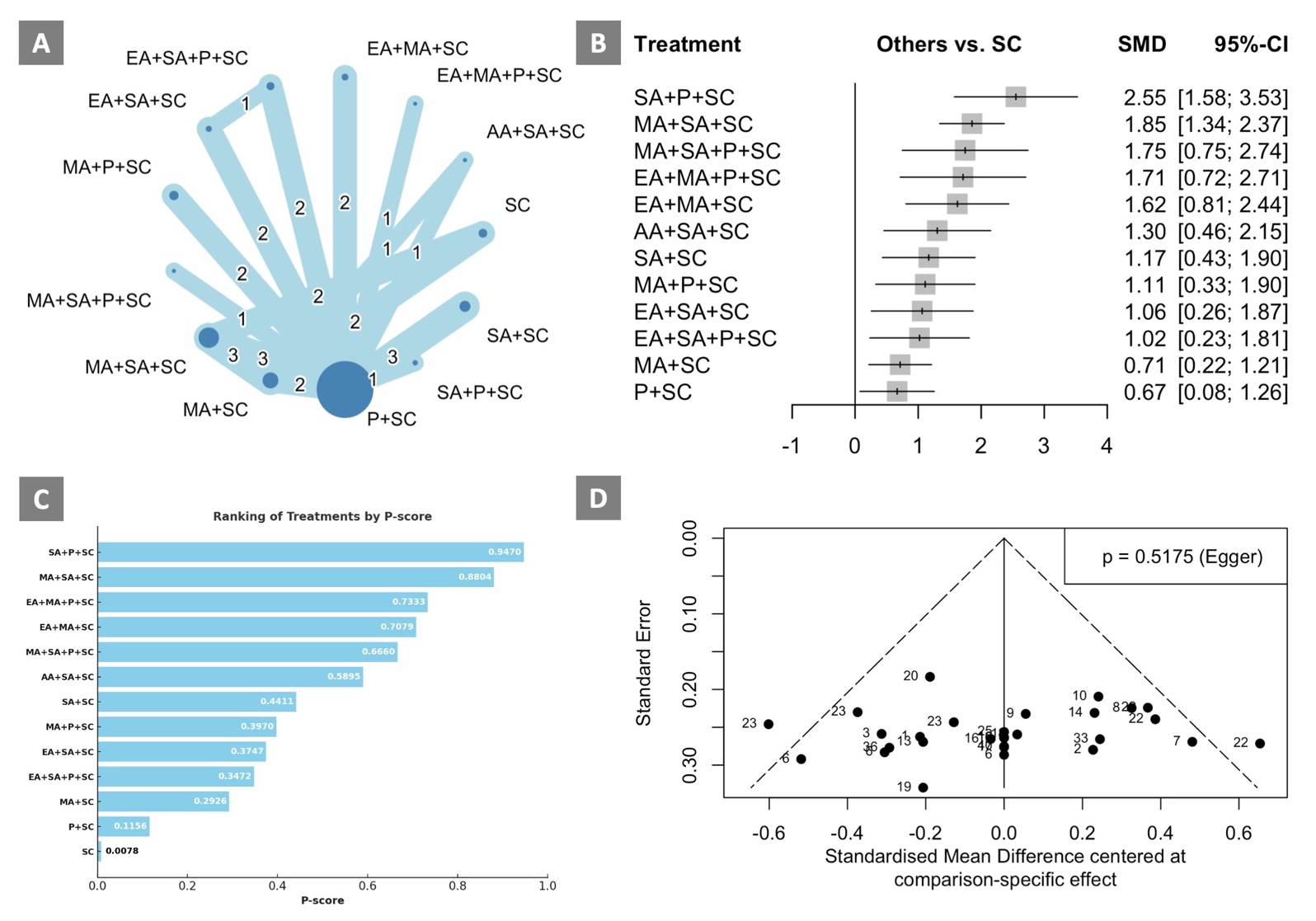
**


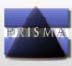
 **PRISMA 2020 Checklist**

| **Section and Topic** | **Item #** | **Checklist item** | **Location where item is reported** |
| --- | --- | --- | --- |
| **TITLE** | | |  |
| Title | 1 | Identify the report as a systematic review. | p.1 |
| **ABSTRACT** | | |  |
| Abstract | 2 | See the PRISMA 2020 for Abstracts checklist. | p.2 |
| **INTRODUCTION** | | |  |
| Rationale | 3 | Describe the rationale for the review in the context of existing knowledge. | p.4 |
| Objectives | 4 | Provide an explicit statement of the objective(s) or question(s) the review addresses. | p.5 |
| **METHODS** | | |  |
| Eligibility criteria | 5 | Specify the inclusion and exclusion criteria for the review and how studies were grouped for the syntheses. | p.6 |
| Information sources | 6 | Specify all databases, registers, websites, organisations, reference lists and other sources searched or consulted to identify studies. Specify the date when each source was last searched or consulted. | p.6 |
| Search strategy | 7 | Present the full search strategies for all databases, registers and websites, including any filters and limits used. | p.6 |
| Selection process | 8 | Specify the methods used to decide whether a study met the inclusion criteria of the review, including how many reviewers screened each record and each report retrieved, whether they worked independently, and if applicable, details of automation tools used in the process. | p.6 |
| Data collection process | 9 | Specify the methods used to collect data from reports, including how many reviewers collected data from each report, whether they worked independently, any processes for obtaining or confirming data from study investigators, and if applicable, details of automation tools used in the process. | p.6 |
| Data items | 10a | List and define all outcomes for which data were sought. Specify whether all results that were compatible with each outcome domain in each study were sought (e.g. for all measures, time points, analyses), and if not, the methods used to decide which results to collect. | p.7 |
|  | 10b | List and define all other variables for which data were sought (e.g. participant and intervention characteristics, funding sources). Describe any assumptions made about any missing or unclear information. | p.7 |
| Study risk of bias assessment | 11 | Specify the methods used to assess risk of bias in the included studies, including details of the tool(s) used, how many reviewers assessed each study and whether they worked independently, and if applicable, details of automation tools used in the process. | p.6 |
| Effect measures | 12 | Specify for each outcome the effect measure(s) (e.g. risk ratio, mean difference) used in the synthesis or presentation of results. | p.7 |
| Synthesis methods | 13a | Describe the processes used to decide which studies were eligible for each synthesis (e.g. tabulating the study intervention characteristics and comparing against the planned groups for each synthesis (item #5)). | p.7 |
|  | 13b | Describe any methods required to prepare the data for presentation or synthesis, such as handling of missing summary statistics, or data conversions. | p.8 |
|  | 13c | Describe any methods used to tabulate or visually display results of individual studies and syntheses. | p.8 |
|  | 13d | Describe any methods used to synthesize results and provide a rationale for the choice(s). If meta-analysis was performed, describe the model(s), method(s) to identify the presence and extent of statistical heterogeneity, and software package(s) used. | p.8 |
|  | 13e | Describe any methods used to explore possible causes of heterogeneity among study results (e.g. subgroup analysis, meta-regression). | p.9 |
|  | 13f | Describe any sensitivity analyses conducted to assess robustness of the synthesized results. | p.9 |
| Reporting bias assessment | 14 | Describe any methods used to assess risk of bias due to missing results in a synthesis (arising from reporting biases). | p.9 |
| Certainty assessment | 15 | Describe any methods used to assess certainty (or confidence) in the body of evidence for an outcome. | p.10 |
| **RESULTS** | | |  |
| Study selection | 16a | Describe the results of the search and selection process, from the number of records identified in the search to the number of studies included in the review, ideally using a flow diagram. | p.10 |
|  | 16b | Cite studies that might appear to meet the inclusion criteria, but which were excluded, and explain why they were excluded. | p.11 |
| Study characteristics | 17 | Cite each included study and present its characteristics. | p.34 |
| Risk of bias in studies | 18 | Present assessments of risk of bias for each included study. | p.17 |
| Results of individual studies | 19 | For all outcomes, present, for each study: (a) summary statistics for each group (where appropriate) and (b) an effect estimates and its precision (e.g. confidence/credible interval), ideally using structured tables or plots. | p.36 |
| Results of syntheses | 20a | For each synthesis, briefly summarise the characteristics and risk of bias among contributing studies. | p.17 |
|  | 20b | Present results of all statistical syntheses conducted. If meta-analysis was done, present for each the summary estimate and its precision (e.g., confidence/credible interval) and measures of statistical heterogeneity. If comparing groups, describe the direction of the effect. | p.14 |
|  | 20c | Present results of all investigations of possible causes of heterogeneity among study results. | p.14 |
|  | 20d | Present results of all sensitivity analyses conducted to assess the robustness of the synthesized results. | p.12 |
| Reporting biases | 21 | Present assessments of risk of bias due to missing results (arising from reporting biases) for each synthesis assessed. | p.17 |
| Certainty of evidence | 22 | Present assessments of certainty (or confidence) in the body of evidence for each outcome assessed. | p.17 |
| **DISCUSSION** | | |  |
| Discussion | 23a | Provide a general interpretation of the results in the context of other evidence. | p.18 |
|  | 23b | Discuss any limitations of the evidence included in the review. | p.19 |
|  | 23c | Discuss any limitations of the review processes used. | p.20 |
|  | 23d | Discuss implications of the results for practice, policy, and future research. | p.21 |
| **OTHER INFORMATION** | | |  |
| Registration and protocol | 24a | Provide registration information for the review, including register name and registration number, or state that the review was not registered. | p.6 |
|  | 24b | Indicate where the review protocol can be accessed, or state that a protocol was not prepared. | p.6 |
|  | 24c | Describe and explain any amendments to information provided at registration or in the protocol. | p.6 |
| Support | 25 | Describe sources of financial or non-financial support for the review, and the role of the funders or sponsors in the review. | p.23 |
| Competing interests | 26 | Declare any competing interests of review authors. | p.23 |
| Availability of data, code and other materials | 27 | Report which of the following are publicly available and where they can be found template data collection forms; data extracted from included studies; data used for all analyses; analytic code; any other materials used in the review. | p.23 |
